# m^6^A demethylase ALKBH5 is required for antibacterial innate defense by intrinsic motivation of neutrophil migration

**DOI:** 10.1038/s41392-022-01020-z

**Published:** 2022-06-29

**Authors:** Yang Liu, Renjie Song, Lu Zhao, Zhike Lu, Yini Li, Xinyi Zhan, Fengjiao Lu, Jiang Yang, Yamei Niu, Xuetao Cao

**Affiliations:** 1 Department of Immunology, Institute of Basic Medical Sciences, Peking Union Medical College, Chinese Academy of Medical Sciences, 100005 Beijing, China; 2 Frontier Research Center for Cell Response, Institute of Immunology, College of Life Sciences, Nankai University, 300071 Tianjin, China; 3 School of Life Sciences, Westlake University, 310024 Hangzhou, China; 4 Department of Pathology, Institute of Basic Medical Sciences, Peking Union Medical College, Chinese Academy of Medical Sciences, 100005 Beijing, China

**Keywords:** Innate immunity, Inflammation

## Abstract

Neutrophil migration into the site of infection is necessary for antibacterial innate defense, whereas impaired neutrophil migration may result in excessive inflammation and even sepsis. The neutrophil migration directed by extracellular signals such as chemokines has been extensively studied, yet the intrinsic mechanism for determining neutrophil ability to migrate needs further investigation. *N*^*6*^-methyladenosine (m^6^A) RNA modification is important in immunity and inflammation, and our preliminary data indicate downregulation of RNA m^6^A demethylase alkB homolog 5 (ALKBH5) in neutrophils during bacterial infection. Whether m^6^A modification and ALKBH5 might intrinsically modulate neutrophil innate response remain unknown. Here we report that ALKBH5 is required for antibacterial innate defense by enhancing intrinsic ability of neutrophil migration. We found that deficiency of ALKBH5 increased mortality of mice with polymicrobial sepsis induced by cecal ligation and puncture (CLP), and *Alkbh5*-deficient CLP mice exhibited higher bacterial burden and massive proinflammatory cytokine production in the peritoneal cavity and blood because of less neutrophil migration. *Alkbh5*-deficient neutrophils had lower CXCR2 expression, thus exhibiting impaired migration toward chemokine CXCL2. Mechanistically, ALKBH5-mediated m^6^A demethylation empowered neutrophils with high migration capability through altering the RNA decay, consequently regulating protein expression of its targets, neutrophil migration-related molecules, including increased expression of neutrophil migration-promoting CXCR2 and NLRP12, but decreased expression of neutrophil migration-suppressive PTGER4, TNC, and WNK1. Our findings reveal a previously unknown role of ALKBH5 in imprinting migration-promoting transcriptome signatures in neutrophils and intrinsically promoting neutrophil migration for antibacterial defense, highlighting the potential application of targeting neutrophil m^6^A modification in controlling bacterial infections.

## Introduction

Neutrophils, a major component of innate immune response and dominant population of leukocytes in the peripheral blood, are essential for host innate defense against invading pathogens. During bacterial challenge, large amounts of neutrophils are mobilized and released from the bone marrow into the peripheral circulation and migrate to the sites of infection at a very early stage.^1,2^ Rapid and effective neutrophil migration and accumulation in the infection site is critical for antibacterial innate defense and subsequent inflammation resolution. The migration and function of neutrophils can be orchestrated by the extracellular signals such chemokines and cytokines, or by neutrophil intrinsic elements including chemokine receptors, cytoskeletal proteins, intracellular signaling mode, and cellular metabolism.^3–7^ As the first line of innate response, once neutrophil migration is impaired due to dysregulated processes above, the host cannot timely launch effective innate defense against bacterial infection, which may result in failure in bacterial clearance and lead to uncontrolled systemic inflammation and even sepsis. In other side, bacterial infection may hijack neutrophil migration to escape the host innate defense, contributing to persistent infections and unresolved inflammation.^5,8^ Therefore, identifying new intracellular molecules that intrinsically determine the migratory ability of neutrophils will, once properly targeted to activate, not only enhance antibacterial innate defense at the early stage of infection, but also appropriately induce the inflammation resolution to avoid tissue damage after the elimination of invading bacteria. Up to now, the extracellular signals for motivating and recruiting neutrophils have been well studied. However, the mechanisms for intrinsically determining neutrophil migration need to be further investigated.

Innate immune cells, including neutrophils, possess their own specific phenotypes and unique functions. It is well known that the cell-specific gene and protein expression patterns play essential roles in cellular phenotype determination and functional transformation, and the biological processes are regulated at multiple levels from gene transcription, post-transcription, to translation and post-translation.^9^ What are the key mechanisms for making innate immune cells with unique phenotypes and functions need to be fully understood. One critical question arises about the importance of epigenetic modifiers in determining neutrophil differentiation, behavior, and function during innate response and inflammation, or in conferring their cell type-specific responses to bacteria components, which also need to be well identified. Indeed, which epigenetic factor can intrinsically endow neutrophils, especially at the post-transcriptional level, with the ability to efficiently migrate to the site of infection remains unknown.

*N*^*6*^-methyladenosine (m^6^A) RNA modification, as one type of epigenetic modifications, is the most abundant internal modification in mammalian mRNA and has been implicated in a variety of physiological and pathological processes.^10^ The methylation of m^6^A is mainly deposited by the m^6^A methyltransferases such as methyltransferase like 3 (METTL3) and methyltransferase like 14 (METTL14). The m^6^A demethylases ALKBH5 and fat mass and obesity associated (FTO) are responsible for removing m^6^A methylation.^11^ m^6^A RNA modification is extensively involved in immunity and inflammation. Emerging evidences show that the m^6^A modification and its methyltransferases are able to regulate the differentiation and function of innate and adaptive immune cells, such as dendritic cells (DCs), macrophages, NK cells, and T cells. For example, METTL3-mediated m^6^A modification increases translation of certain immune transcripts for physiological promotion of DC activation and DC-based T cell response.^12^ Besides, METTL3 controls T cell homeostasis and differentiation by enhancing decay of *Socs* gene family mRNA in T cells upon IL-7 signaling.^13^ The expression of METTL3 in tumor-infiltrating NK cells is decreased, which contributes to the impaired NK cell infiltration and function in the tumor microenvironment.^14^ Yet the effect of m^6^A RNA demethylases on immune cells is largely unexplored. We previously reported that m^6^A RNA demethylase ALKBH5 can rewire α-ketoglutarate dehydrogenase (OGDH)-itaconate metabolism to promote viral replication in macrophages.^15^ Recent study revealed a specific function of ALKBH5 in controlling CD4^+^ T cell-mediated pathogenesis in autoimmunity.^16^ However, the intrinsic role of m^6^A RNA modification and ALKBH5 in neutrophils in antibacterial immunity is still unknown.

Inspired by our preliminary observation on the downregulation of ALKBH5 in neutrophils during bacterial infection, in this study, we investigated the roles of ALKBH5 and m^6^A RNA modification in intrinsically regulating neutrophil behavior in antibacterial innate defense.

## Results

### ALKBH5 is required for restraining bacterial infection and excessive inflammation

**Fig. 1:**
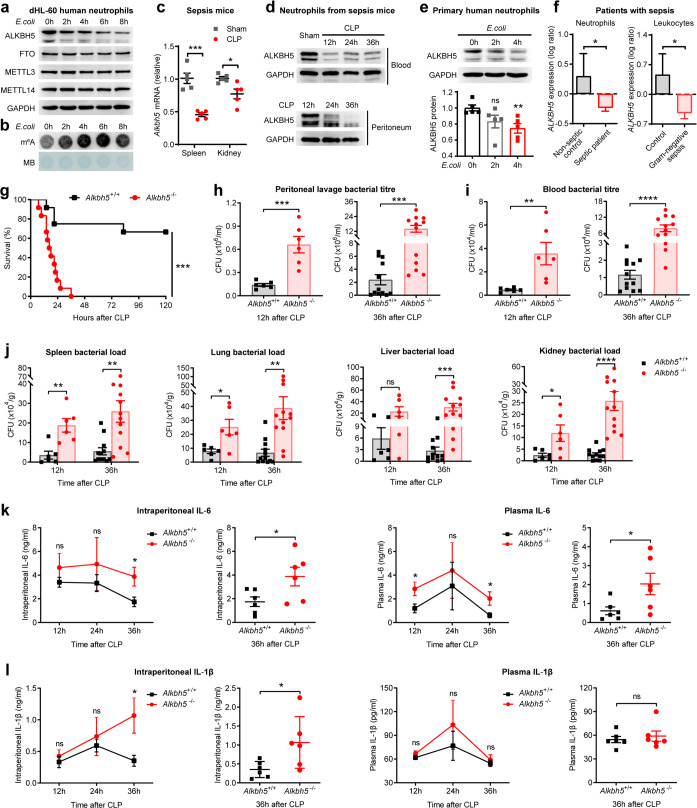
Deficiency of ALKBH5 impairs bacterial clearance and leads to excessive inflammation. **a** Immunoblot analysis of protein levels of indicated m^6^A enzymes in the differentiated HL-60 neutrophil-like cells (dHL-60) infected with *E.coli* for the indicated times. **b** m^6^A dot blot assay of global RNA m^6^A abundance in dHL-60 cells as in **a**. MB, methylene blue staining (as loading control). **c** qRT-PCR analysis of *Alkbh5* mRNA expression in spleen and kidney of mild cecal ligation and puncture (CLP) or sham- operated wild-type (WT) mice (*n* = 5). qRT-PCR data were normalized to *Gapdh* expression. **d** Immunoblot analysis of protein levels of mouse ALKBH5 in neutrophils from peripheral blood (up) or peritoneal fluid (down) of sham-operated WT controls or WT mice given mild CLP as indicated times. GAPDH, glyceraldehyde-3-phosphate dehydrogenase. **e** Immunoblot analysis of protein levels of human ALKBH5 in primary human neutrophils from healthy donors infected with *E.coli* for the indicated times. The ALKBH5 protein expression intensity (relative to 0 h; bottom) was determined using the ImageJ program (*n* = 5). **f** Comparison of the expression levels of *ALKBH5* in: neutrophils from peripheral blood of septic patients (*n* = 62) or non-septic control patients (*n* = 20) based on the gene expression profiling datasets GSE5772 (left); or leukocytes from Critically ill patients with Gram-negative sepsis (*n* = 25) or controls (*n* = 17) based on the gene expression profiling datasets GSE6535 (right). **g** Survival of *Alkbh5*-deficient mice and WT littermates given a lethal form of CLP. Kaplan–Meier survival curves were compared using Log-rank (Mantel–Cox) test (*n* = 12). **h**, **i** Bacterial titer (CFU) in the peritoneal lavage fluid (**h**) and in the blood (**i**) of *Alkbh5*-deficient mice and WT littermates at indicated times after mild CLP (*n* = 6 for 12 h; *n* = 12 for 36 h). CFU, colony-forming unit. **j** Bacterial loads in the spleen, lungs, liver, and kidney of CLP mice as in **h**, **i**. **k**, **l** ELISA of IL-6 (**k**) and IL-1β (**l**) concentrations in the peritoneal lavage fluid or plasma of *Alkbh5*-deficient mice and WT littermates at indicated times after mild CLP (*n* = 6). IL-6, interleukin 6; IL-1β, interleukin 1 beta. All data are mean ± SEM of biologically independent samples. Data are representative of 3–5 independent experiments with similar results [(**a**, **b**) and (**d**, **e**)]. *P < 0.05; **P < 0.01; ***P < 0.001; ****P < 0.0001; ns not significant. Kaplan–Meier (**g**) or two-tailed unpaired Student’s *t* test [(**c**), (**e**, **f**), and (**h**–**l**)]

Firstly, we analyzed the expression of ALKBH5 and other m^6^A modulators during bacterial infection. In the differentiated HL-60 neutrophil-like (dHL-60) cells infected with *Escherichia coli* (*E.coli*), a prevalent species responsible for sepsis caused by Gram-negative bacteria,^3,17^ we observed declined protein expression of ALKBH5 whereas the unchanged protein levels of other m^6^A enzymes (including FTO, METTL3, and METTL14) upon bacterial infection (Fig. 1a and Supplementary Fig. 1a). Accordingly, there were significantly increased global RNA m^6^A modification levels in dHL-60 human neutrohils upon *E.coli* infection (Fig. 1b), accompanying with the attenuated activity of RNA m^6^A demethylation in cells due to down-regulated protein levels of m^6^A demethylase ALKBH5. These results indicated that ALKBH5 and it-mediated RNA m^6^A demethylation might regulate neutrophil behavior or function during bacterial infection. By subjecting mice to cecal ligation and puncture (CLP), a well-established model of abdominal polymicrobial bacterial infection that is most relevant for clinical sepsis,^4,18^ we found down-regulated mRNA expression of *Alkbh5* in spleen, kidney, and brain of CLP mice (Fig. 1c and Supplementary Fig. 1b) and significant lower protein levels of ALKBH5 in neutrophils isolated from peripheral blood and peritoneal fluid of sepsis mice than that from sham-operated controls (Fig. 1d). Mice injected with *Staphylococcus aureus* display a decreased *Alkbh5* level in whole-blood than uninfected mice (GSE38531) (Supplementary Fig. 1c). Besides, the subcellular localization of ALKBH5, mainly in the nucleus of neutrophils, remained unchanged with or without *E.coli* infection (Supplementary Fig. 1d).

ALKBH5 is highly conserved between human and mouse.^19^ By using primary human neutrophils from healthy donors, we observed a significant decline in protein expression of ALKBH5 upon bacterial infection (Fig. 1e). These data further confirmed that ALKBH5 is down-regulated in both mouse and human neutrophils during bacterial infection, inspiring us to search public database for analyzing ALKBH5 expression in patients with bacterial infectious diseases such as sepsis. The analysis of the gene expression profiling datasets of a sepsis cohort (GSE5772) revealed that *ALKBH5* is expressed at a lower level in neutrophils from peripheral blood of septic patients compared to that from non-septic control patients (Fig. 1f, left). Similarly, leukocytes from Critically ill patients with Gram-negative sepsis have a reduced expression of *ALKBH5* (Fig. 1f, right), as showed in another sepsis cohort (GSE6535). These data are consistent with our observations on ALKBH5 downregulation in neutrophils upon bacterial infection or from sepsis mice. Therefore, the lower expression of ALKBH5 could be a risk hallmark in bacterial infectious diseases, and also might be associated with impaired antibacterial innate defense.

To investigate the effect of downregulated ALKBH5 in antibacterial host defense in vivo, we subjected *Alkbh5*-deficient mice^15,20^ and their wild-type (WT) littermates to CLP. Approximately 50% cecum ligation (mild CLP) can induce mid-grade sepsis, and ~75% cecum ligation-induced high-grade (lethal) sepsis is only used for testing survival.^21^ We found that *Alkbh5*-deficient mice had significantly increased mortality compared to their WT littermates when performed in a lethal form of CLP (Fig. 1g). In a mid-grade sepsis, the in vivo bacterial titer in the peritoneal lavage fluid (Fig. 1h) and blood (Fig. 1i) of *Alkbh5*-deficient mice markedly increased, especially at the late stage of sepsis. Consistent with these observations, *Alkbh5* depletion led to a higher bacterial load in the spleen, lungs, liver, and kidney of CLP mice (Fig. 1j), revealing a crucial role for ALKBH5 in overall controlling of bacterial infection. Besides, the levels of proinflammatory cytokines interleukin 6 (IL-6) and interleukin 1 beta (IL-1β) were higher during the resolution of infection, in peritoneal cavity or plasma of *Alkbh5*-deficient mice than WT littermates after a mild CLP (Fig. 1k, l). Therefore, *Alkbh5*-deficient CLP mice exhibited higher mortality, uncleared bacteria and uncontrolled inflammation, suggesting that ALKBH5 is necessary for effective bacterial clearance and preventing excessive inflammation in vivo.

### ALKBH5 facilitates neutrophil accumulation in the site of infection in antibacterial defense

**Fig. 2:**
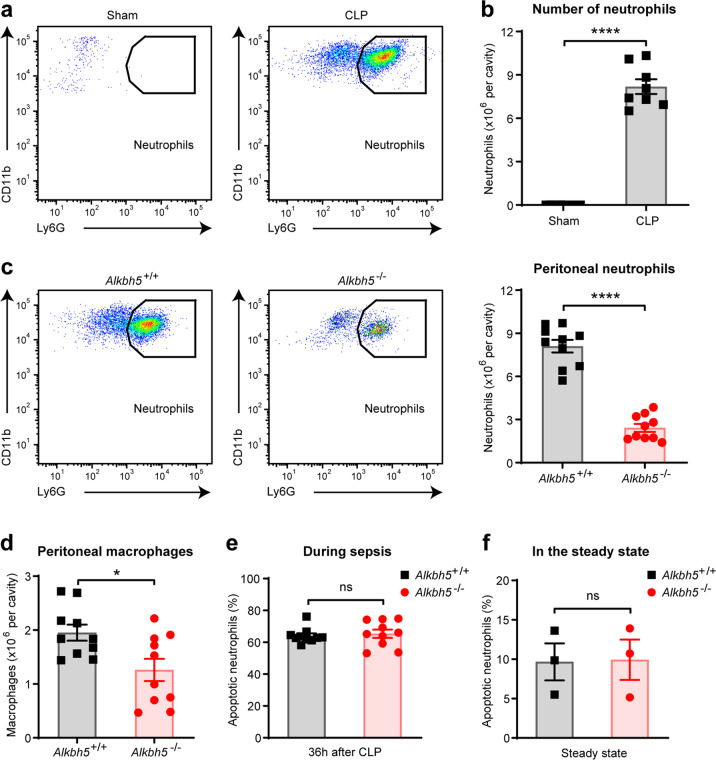
ALKBH5 facilitates neutrophil accumulation in the site of infection in antibacterial defense. **a** FACS analysis of neutrophils in the peritoneal cavity of WT mice operated sham or mild CLP for 36 h. **b** Absolute numbers of peritoneal neutrophils from mice as in (**a**) (*n* = 8). **c** FACS analysis of neutrophils in the peritoneal cavity of *Alkbh5*-deficient mice and WT littermates at 36 h after mild CLP (*n* = 10). **d** FACS analysis of macrophages in the peritoneal cavity of mice as in (**c**) (*n* = 10). **e** FACS analysis of apoptotic neutrophils in the peritoneal cavity of mice as in (**c**) (*n* = 10). **f** FACS analysis of apoptotic neutrophils in the bone marrow of *Alkbh5*-deficient mice and WT littermates in the steady state (*n* = 3). All data are mean ± SEM of biologically independent samples. Data are representative of 8–10 independent experiments with similar results [(**a**) and (**c**)]. *P < 0.05; ****P < 0.0001; ns, not significant. Two-tailed unpaired Student’s *t* test (**b**–**f**)

How does ALKBH5 promote antibacterial defense for bacterial clearance? Neutrophils are the main type of immune cells that first migrate into the sites of infection upon bacterial infection. Successful elimination of invading bacteria depends on efficient neutrophil accumulation in infection sites.^2,22,23^ As expected, FACS analysis showed that neutrophils flowed to the peritoneal cavity as part of the host defense in wild-type CLP mice, and accumulated in this infection site during sepsis (Fig. 2a and Supplementary Fig. 2a). Neutrophils also represented the major immune cell population in the peritoneal cavity of wild-type mice undergoing sepsis, as their numbers significantly increased as compared to sham-operated controls (Fig. 2b). During sepsis, *Alkbh5*-deficient mice exhibited substantially reduced neutrophils in the peritoneal fluid than their WT littermates (Fig. 2c), suggesting that ALKBH5 is required for protective neutrophil accumulation in the site of infection.

Bacteria-induced inflammation and sepsis can cause neutrophil apoptosis and subsequent efferocytosis of apoptotic neutrophils by macrophages, which may result in decrease of neutrophil accumulation and contribute to prevention of excessive inflammation at the late stage of infection.^6,24^ Accompanying with the decreased numbers of peritoneal neutrophils, there was also a modest reduction in the numbers of macrophages in the peritoneal cavity of *Alkbh5*-deficient mice than in WT littermates 36 h after mild CLP (Fig. 2d and Supplementary Fig. 2b). Meanwhile, we observed no difference in the frequency of apoptotic neutrophils in the peritoneal cavity of *Alkbh5*-deficient mice when compared with WT littermates undergoing sepsis (Fig. 2e and Supplementary Fig. 2c). Besides, *Alkbh5* deletion did not affect the count or apoptosis of neutrophils in bone marrow of mice under steady state (Fig. 2f and Supplementary Fig. 3a–c). These data suggest that apoptosis of neutrophils or macrophage-mediated efferocytosis are not the cause of reduced peritoneal neutrophils in *Alkbh5*-deficient CLP mice. Therefore, ALKBH5 promotes neutrophil accumulation in the site of infection for bacterial clearance.

### ALKBH5 improves the migratory ability of neutrophils

**Fig. 3:**
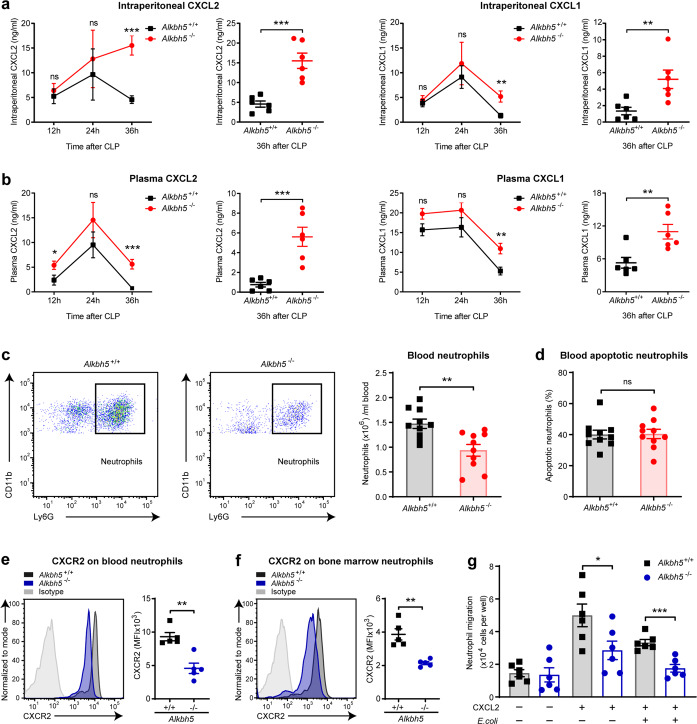
Deficiency of ALKBH5 reduces the migratory ability of neutrophils toward chemokines. **a**, **b** ELISA of CXCL2 and CXCL1 concentrations in the peritoneal lavage fluid (**a**) and plasma (**b**) of *Alkbh5*-deficient mice and WT littermates at indicated times after mild CLP (*n* = 6). **c** FACS analysis of neutrophils in the blood of *Alkbh5*-deficient mice and WT littermates 36 h after mild CLP (*n* = 10). **d** FACS analysis of apoptotic neutrophils in the blood of mice as in (**c**) (*n* = 10). **e**, **f** FACS analysis of protein expression of CXCR2 on cell-surface of neutrophils from blood (**e**) or bone marrow (**f**) of *Alkbh5*-deficient mice and WT littermates 24 h after mild CLP (*n* = 5). MFI, mean fluorescence intensity. **g** Transwell migration assay of neutrophil migration toward CXCL2. Neutrophils that purified from the bone marrow of *Alkbh5*-deficient mice and WT littermates at steady state were infected with or without *E.coli* and then treated with CXCL2 (30 ng/ml) for 2 h as indicated, then migration assay was determined (*n* = 6). All data are mean ± SEM of biologically independent samples. Data are representative of 5 or 10 independent experiments with similar results [(**c**) and (**e**, **f**)]. *P < 0.05; **P < 0.01; ***P < 0.001; ns, not significant. Two-tailed unpaired Student’s *t* test (**a**–**g**)

Neutrophil recruitment from bone marrow to infectious tissues is critical for early innate responses. The accumulation of neutrophils in infection site is dependent on their remarkable ability to migrate within and through circulation, which is mainly triggered by interaction between chemokines and chemokine receptors.^5^ Chemokine (C-X-C motif) ligand 2 (CXCL2) and CXCL1 are largely responsible for driving neutrophil migration during bacterial infection and inflammation.^2,6,16^ Interestingly, we found that the levels of CXCL2 and CXCL1 in peritoneal cavity and plasma of *Alkbh5*-deficient mice were not decreased during sepsis, but even higher at 36 h after given mild CLP (the resolution phase of sepsis) (Fig. 3a, b). Yet deficiency of ALKBH5 did not affect the levels of CXCL2 and CXCL1 in plasma, peritoneal cavity, or bone marrow of mice in the steady state (Supplementary Fig. 4a, b). These data excluded the possibility that the impaired neutrophil accumulation was due to decreased neutrophil-attracting chemokines in *Alkbh5*-deficient CLP mice. In addition to the significant decline of neutrophils in peritoneal cavity as mentioned above (Fig. 2c), we also observed lower number of neutrophils in the peripheral blood of *Alkbh5*-deficient mice than in WT littermates 36 h after mild CLP (Fig. 3c and Supplementary Fig. 4c). Meanwhile, there was no significant change in frequency of apoptotic neutrophils in blood of CLP mice upon loss of ALKBH5 (Fig. 3d and Supplementary Fig. 4d), excluding the possibility that the reduction of blood neutrophils in *Alkbh5*-deficient CLP mice was due to increased apoptosis of neutrophils. Together, these data suggest that the intrinsic migratory ability of *Alkbh5*-deficient neutrophils may be impaired, thus resulting in failed migration in response to extracellular chemotactic signals.

Chemokine (C-X-C motif) receptor 2 (CXCR2) is a critical chemokine receptor to be responsible for neutrophil chemotaxis to infection sites, driven by its CXC chemokine ligands such as CXCL2.^2,25,26^ Noticeably, *Alkbh5*-deficient mice exhibited decreased protein expression of CXCR2 on cell surface of neutrophils from blood and bone marrow as compared to that in WT littermates, after mild CLP (Fig. 3e, f). Transwell migration assay confirmed that *Alkbh5*-deficient bone marrow neutrophils exhibited significantly defective migration toward CXCL2 in vitro (Fig. 3g). During CLP-induced sepsis, CCL2 is a critical mediator for effective macrophage recruitment to peritoneal cavity and is mainly derived from peritoneal neutrophils.^27^ Yet deletion of ALKBH5 did not affect macrophage migration directly (Supplementary Fig. 5a, b). Subsequent experiments showed the decreased peritoneal CCL2 levels in CLP mice upon ALKBH5 deletion (Supplementary Fig. 5c), implying that the reduced peritoneal macrophages in *Alkbh5*-deficient CLP mice may be attributed to less local CCL2 production by the reduced neutrophils in peritoneal cavity. Taken together, these results indicate that ALKBH5 expression in neutrophils directly endows neutrophils with the potent capability to effectively migrate at least toward chemokines, ensuring neutrophil recruitment from bone marrow to infectious tissues.

### ALKBH5 imprints migration-promoting transcriptome signatures in neutrophils in antibacterial defense

**Fig. 4:**
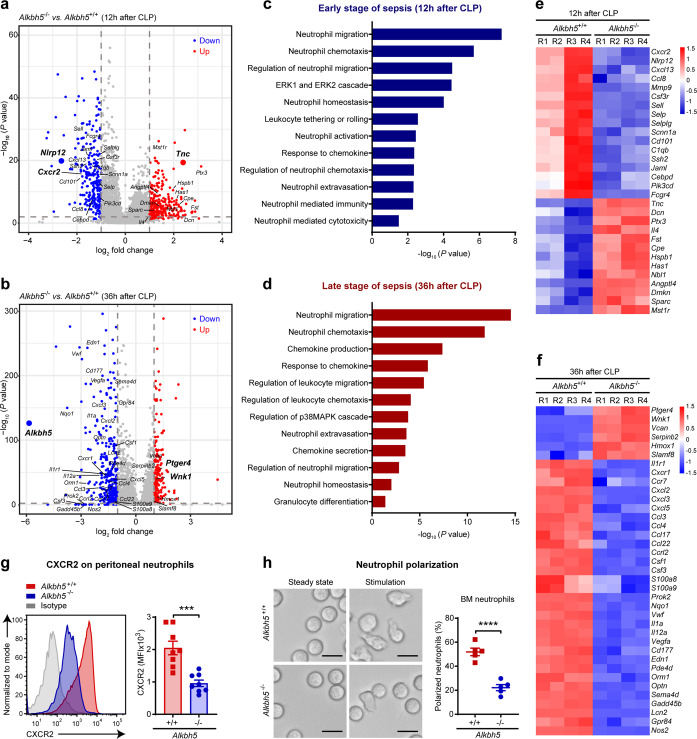
ALKBH5 imprints migration-promoting transcriptome signatures in neutrophils in antibacterial defense. **a**, **b** Volcano plots of gene expression profiles in peritoneal neutrophils from *Alkbh5*-deficient mice (*Alkbh5*^–*/–*^) and WT littermates (*Alkbh5*^*+/+*^) at 12 h (**a**) or 36 h (**b**) after mild CLP, respectively. **a** was zoomed-in partial region of complete volcano plot (For complete volcano plot of CLP 12 h, see Supplementary Fig. S6c). Genes with significant changes in expression upon *Alkbh5* deficiency were colored by red for up-regulated genes and by blue for down-regulated genes. *Alkbh5* and its potential targets were labeled near bigger plots. Four independent biological replicates. **c**, **d** Visualization of the GO biological processes enrichment analysis of the significantly differentially expressed genes (DEGs) upon *Alkbh5* deficiency with annotations of neutrophil association. The early stage of sepsis showed significantly DEGs at 12 h after mild CLP (**c**); the late stage of sepsis showed significantly DEGs at 36 h after mild CLP (**d**). **e**, **f** Heatmap showing the expression variations of the DEGs (related to neutrophil migration and chemotaxis) in peritoneal neutrophils from *Alkbh5*-deficient mice (*Alkbh5*^*–/–*^) versus WT littermates (*Alkbh5*^*+/+*^) at 12 h (**e**) or 36 h (**f**) after mild CLP, respectively. Four biological replicates (R1 to R4). **g** FACS analysis of protein expression of CXCR2 on cell-surface of peritoneal neutrophils from *Alkbh5*-deficient mice and WT littermates at 12 h after mild CLP (*n* = 8). **h** Polarization assay of bone marrow neutrophils from normal *Alkbh5*-deficient mice and WT littermates. Scale bars, 10 μm. The percentage of polarized neutrophils (cells that ruffled or extended pseudopods) was calculated after *E.coli* and CXCL2 stimulation (*n* = 5). All data are mean ± SEM of biologically independent samples. Data are representative of 5 or 8 independent experiments with similar results (**g, h**). ***P < 0.001; ****P < 0.0001. Two-tailed unpaired Student’s *t* test (**g, h**)

Next we went further to investigate how ALKBH5 intrinsically improves the migratory ability of neutrophils, for examples, how to upregulate CXCR2 protein expression on neutrophils and what other intracellular melocules targeted by ALKBH5 for endowing neutrophil migration? We performed transcriptome-wide RNA sequencing (RNA-seq) on neutrophils isolated from the peritoneal cavity of *Alkbh5*-deficient mice and WT littermates during early stage of sepsis (12 h after mild CLP) or late stage of sepsis (36 h after mild CLP), respectively. Four biological replicates correlated strongly with each other, representing good reproducibility and reliability of data (Supplementary Fig. 6a, b). RNA-seq analysis showed that *Alkbh5* deficiency in neutrophils resulted in up-regulation of 297 genes and down-regulation of 264 genes at the early stage of sepsis (Fig. 4a and Supplementary Fig. 6c), while up-regulation of 197 genes and down-regulation of 337 genes at the late stage of sepsis (Fig. 4b). A specific absence of *Alkbh5* mRNA in *Alkbh5*^–/–^ versus *Alkbh5*^+/+^ groups also confirmed efficient deletion of *Alkbh5* gene in *Alkbh5*-deficient neutrophils in our system (Fig. 4b and Supplementary Fig. 6c).

Gene Ontology (GO) biological processes enrichment analysis of the significantly differentially expressed genes (DEGs) in *Alkbh5*-deficient neutrophils compared with WT neutrophils showed that neutrophil migration made up the most significantly enriched biological processes with annotations of neutrophil association upon deletion of ALKBH5 at both the early and late stages of sepsis (Fig. 4c, d). Many significantly DEGs also encompassed transcriptional signatures related to neutrophils, specifically to neutrophil influx into the infection site, including chemotaxis, response to chemokine, extravasation, ERK1 and ERK2 cascade, and neutrophil homeostasis (Fig. 4c, d). During the late stage of sepsis, some varied genes were enriched for other neutrophil migration-associated processes such as chemokine production and p38MAPK pathway (Fig. 4d). Notably, ALKBH5 deletion led to significant suppression of *Cxcr2* transcripts in neutrophils (Fig. 4e), which was consistent with our in vivo observation about decreased CXCR2 protein expression on *Alkbh5*-deficient peritoneal neutrophils during early stage of sepsis (Fig. 4g and Supplementary Fig. 7a).

Then, we focused on the differentially expressed genes that are critical for neutrophil migration (Fig. 4e, f). During the early stage of sepsis, lack of ALKBH5 significantly down-regulated the genes involved in promotion of neutrophil migration, such as NLR family pyrin domain containing 12 (*Nlrp12*) and matrix metallopeptidase 9 (*Mmp9*), in neutrophils; Conversely, ALKBH5 deletion increased mRNA expression of some inhibitory molecules for neutrophil migration such as tenascin C (*Tnc*) and *Ptx3* (Fig. 4e). At the late stage of sepsis, the transcript levels of several neutrophil migration inhibitors, such as prostaglandin E receptor 4 (*Ptger4*), WNK lysine deficient protein kinase 1 (*Wnk1*), and *Vcan*, were significantly increased; while the transcript levels of multiple cytokine and chemokine receptors (such as *Il1r1*, *Cxcr1*, and *Ccr7*) and neutrophil-recruiting chemokines and proinflammatory factors (including *Cxcl2*, *Cxcl3*, *Cxcl5*, *Il1a*, *Prok2*, *Csf3*, *Vegfa*, *S100a8*, and *S100a9*)^1,6^ were decreased, in neutrophils from *Alkbh5*-deficient CLP mice (Fig. 4f). These above potential ALKBH5-targeted genes, which are involved in neutrophil migration, further highlight the importance of ALKBH5 in endowing neutrophil migration in innate defense.

Neutrophil migration requires the coordination of signaling pathways at the front and rear of cell that lead to neutrophil polarization and motility, which is characterized by a polarized morphology.^3^ Neutrophils from bone marrow of normal mice were predominantly round in the steady state, while displayed membrane ruffles and polarized upon chemoattractant stimulation (Fig. 4h). Moreover, *Alkbh5*-deficient neutrophils exhibited an impaired neutrophil polarization compared with WT neutrophils in response to chemoattractant (Fig. 4h). Alongside the changed expression of neutrophil migration-associated genes, a few transcripts related to neutrophil activation pathway were observed to be altered in *Alkbh5-*deficient neutrophils (Fig. 4c). In vitro functional assays, using the same number of bone marrow neutrophils, showed that disruption of ALKBH5 had no direct effect on the phagocytosis or bacteria-killing capability of neutrophils (Supplementary Fig. 7b, c). Therefore, ALKBH5 drives a migration-promoting transcriptional landscape of neutrophils to enable their migration into the site of infection for bacterial eradication.

### ALKBH5 enhances CXCR2 but suppresses migration-inhibitory molecule expression in neutrophils

**Fig. 5:**
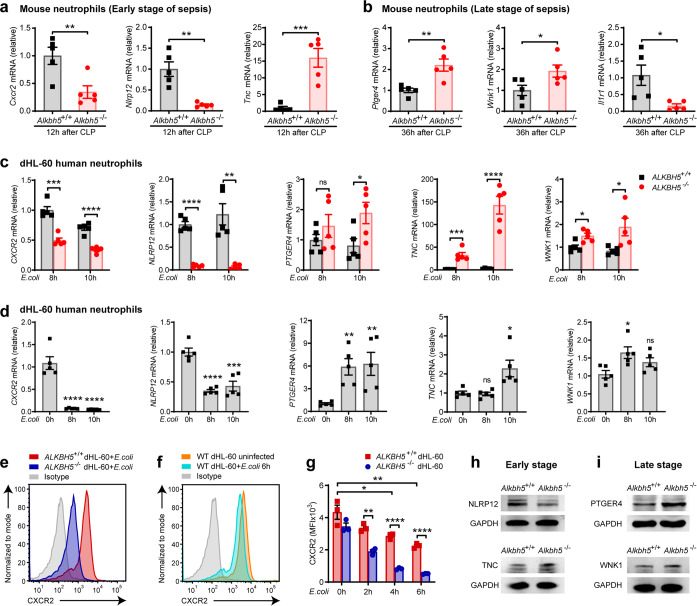
ALKBH5 selectively enhances migration-promoting molecules but suppresses migration-inhibitory genes in neutrophils. **a**, **b** qRT-PCR of the mRNA levels of indicated genes in peritoneal neutrophils isolated from *Alkbh5*-deficient mice (*Alkbh5*^–/–^) and WT littermates (*Alkbh5*^+/+^) at 12 h (**a**) or 36 h (**b**) after mild CLP (*n* = 5). **c** qRT-PCR of the mRNA levels of indicated genes in *ALKBH5*-deficient (*ALKBH5*^–/–^) and WT (*ALKBH5*^+/+^) dHL-60 cells infected with *E.coli* as indicated times (*n* = 5). **d** qRT-PCR of the mRNA levels (relative to 0 h) of indicated genes in WT dHL-60 cells infected with *E.coli* as indicated times (*n* = 5). **e** FACS analysis of the cell-surface protein-level expression of CXCR2 on *ALKBH5*^+/+^ and *ALKBH5*^–/–^ dHL-60 cells infected with *E.coli* for 6 h. **f** FACS analysis of the cell-surface protein-level expression of CXCR2 on WT dHL-60 cells infected with *E.coli* or uninfected as indicated. **g** Quantification of CXCR2 MFI on indicated dHL-60 cells as in (**e**) and (**f**) (*n* = 3). MFI mean fluorescence intensity. **h**, **i** Immunoblot analysis of the protein expression levels of indicated genes in blood neutrophils from *Alkbh5*-deficient mice (*Alkbh5*^–/–^) and WT littermates (*Alkbh5*^+/+^) at 12 h (**h**) or 36 h (**i**) after mild CLP. All data are mean ± SEM of biologically independent samples. qRT-PCR data were normalized to *Gapdh* (**a**, **b**) or *GAPDH* (**c**, **d**) expression. Data are representative of 3 independent experiments with similar results [(**e**, **f**) and (**h**, **i**)]. *P < 0.05; **P < 0.01; ***P < 0.001; ****P < 0.0001; ns not significant. Two-tailed unpaired Student’s *t* test [(**a**–**d**) and (**g**)]

In order to identify the downstream targets of ALKBH5 involved in neutrophil migration, we then confirmed the ALKBH5-driven transcriptional programs in neutrophils. In addition to *Cxcr2*, among those significantly differentially expressed genes indicated by RNA-seq data, another two critical intrinsic regulators of neutrophil migration, *Nlrp12* and *Ptger4* (also known as *EP4*), were substantially down- or up- regulated in *Alkbh5*-deficient neutrophils respectively (Fig. 4e, f). The innate sensor NLRP12 promotes neutrophil migration in innate defense,^28,29^ and *Nlrp12*-deficient neutrophils fail to respond to chemokines,^30^ indicating a cell-intrinsic role for NLRP12 in licensing neutrophil migration. Activation of G-protein coupled receptor family member PTGER4 can inhibit neutrophil migration to the inflamed sites of mice.^31–33^ qRT-PCR verification revealed that deletion of ALKBH5 significantly down-regulated the mRNA expression of *Cxcr2* and *Nlrp12*, while up-regulated the *Ptger4* mRNA level in peritoneal neutrophils from mice at the early or late stage of sepsis (Fig. 5a, b). In addition, RNA-seq analysis showed that neutrophils lack of ALKBH5 exhibited up-regulated mRNA expression of two neutrophil migration-suppressors *Tnc* and *Wnk1*, whereas decreased mRNA level of *Il1r1* (Fig. 4e, f). The extracellular matrix protein TNC inhibits neutrophil chemotaxis.^34^ WNK1 decreases neutrophil accumulation in the peritoneum of mice.^35^ There were increased mRNA levels of *Tnc* and *Wnk1*, reduced *Il1r1* transcripts in peritoneal neutrophils from *Alkbh5*-deficient mice than WT littermates undergoing sepsis (Fig. 5a, b).

Next we confirmed these potential target genes of ALKBH5 in human neutrophils through generation of *ALKBH5* knockout HL-60 cell line via CRISPR-Cas9 technology (Supplementary Fig. 7d, e). *ALKBH5* deficiency indeed reduced the mRNA expression of *CXCR2* and *NLRP12*, while increased mRNA expression of *PTGER4*, *TNC*, and *WNK1* in dHL-60 human neutrophils (Fig. 5c). These findings suggest that these five genes represent downstream targets of ALKBH5 and their expression can be directly regulated by ALKBH5. In addition, *E.coli* infection led to down-regulation of *CXCR2* and *NLRP12*, up-regulation of *PTGER4*, *TNC*, and *WNK1*, in WT dHL-60 human neutrophils (Fig. 5d), which might due to the decreased expression of ALKBH5 protein in *E.coli*-infected neutrophils (Fig. 1).

We then asked whether the protein expression of these targets could be regulated by ALKBH5. The cell-surface expression of CXCR2 protein was significantly decreased on *ALKBH5*-deficient dHL-60 human neutrophils infected with *E.coli* (Fig. 5e, g), which was consistent with our in vivo results that loss of ALKBH5 declined CXCR2 protein expression on neutrophils from sepsis mice (Figs. 3e, f and 4g). Moreover, *E.coli* infection also markedly down-regulated the CXCR2 protein levels on WT dHL-60 human neutrophils (Fig. 5f, g), which might be caused by the reduced ALKBH5 protein in *E.coli*-infected neutrophils (Fig. 1a). In accordance with the RNA-seq and qRT-PCR data, deletion of ALKBH5 decreased NLRP12, while increased TNC protein expression in neutrophils during the early stage of infection (Fig. 5h). During the late stage of infection, higher protein levels of PTGER4 and WNK1 were detected in *Alkbh5*-deficient neutrophils (Fig. 5i). Therefore, ALKBH5 directly modulates the expression of these neutrophil migration-related genes in both mouse and human neutrophils, playing a conserved role in intrinsically promoting neutrophil migration.

### ALKBH5-mediated m^6^A demethylation modulates RNA decay of target molecules for neutrophil migration

**Fig. 6:**
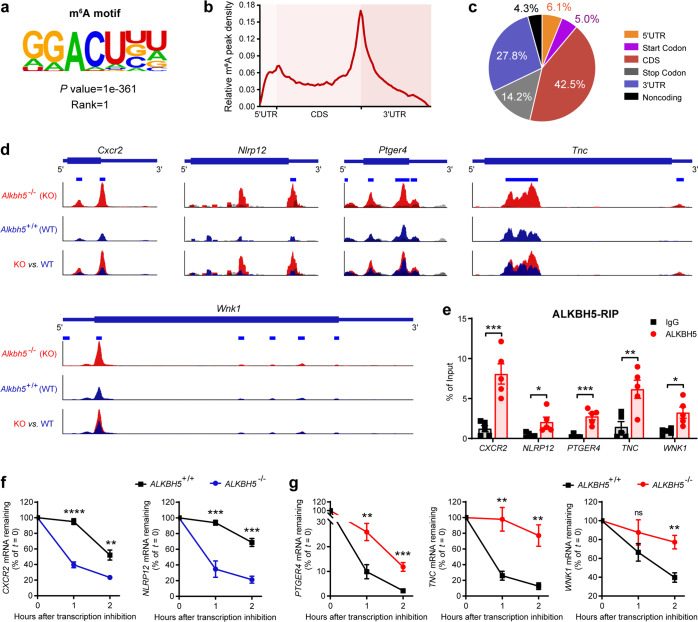
ALKBH5 regulates targets’ mRNA expression via m^6^A demethylation-modulated changes in RNA decay for neutrophil migration. **a** Consensus motifs and *P* value of m^6^A peaks identified by HOMER from m^6^A-seq of dHL-60 human neutrophils infected with *E.coli*. Two independent biological replicates. **b** Metagene profiles of m^6^A peak density along a normalized transcript as in **a**. CDS, protein coding sequence; 3′ UTR, 3′ untranslated region. **c** The distribution of m^6^A peak in the indicated transcript segments as in **a**. **d** m^6^A abundance on indicated mRNAs from our m^6^A-seq data (GSE127732). Red, *Alkbh5*^–/–^ IP; blue, *Alkbh5*^+/+^ IP; gray, input. The y axis represents the normalized m^6^A signal along the gene. Two independent biological replicates. **e**. RIP-qPCR analysis of ALKBH5 binding to indicated mRNAs in dHL-60 human neutrophils infected with *E.coli* (*n* = 5). **f**, **g** mRNA degradation of indicated targets in WT (*ALKBH5*^+/+^) and *ALKBH5*-deficient (*ALKBH5*^–/–^) dHL-60 human neutrophils treated with actinomycin D for the indicated times (*n* = 5). Data were normalized to *18S rRNA* expression and the residual mRNAs were normalized to *t* = 0 h. All data are mean ± SEM of biologically independent samples. *P < 0.05; **P < 0.01; ***P < 0.001; ****P < 0.0001; ns not significant. Two-tailed unpaired Student’s *t* test (**e**–**g**)

As RNA m^6^A demethylase, ALKBH5 post-transcriptionally regulates gene expression in m^6^A modification-dependent manner.^16,36^ To gain insight into the mechanism underlying the effect of ALKBH5 on its targets, we performed transcriptome-wide m^6^A methylation profiling (m^6^A-seq). The consensus m^6^A motifs were most significantly enriched within the m^6^A peaks with typical m^6^A peak distribution features (Fig. 6a, b), and m^6^A methylation sites were located primarily in the protein coding sequence region and 3′ untranslated region of transcripts (Fig. 6c), in neutrophils after bacteria challenge. These m^6^A methylation patterns were consistent with our previous study and other published works.^15,37,38^ Notably, our m^6^A-seq data (GSE127732) revealed that specific m^6^A peaks were clearly enriched on *Cxcr2*, *Nlrp12*, *Ptger4*, *Tnc*, and *Wnk1* mRNAs, which were significantly increased upon loss of ALKBH5 (Fig. 6d and Supplementary Table. 1). Indeed, RNA immunoprecipitation (RIP)-qPCR assay confirmed that the mRNAs of *CXCR2*, *NLRP12*, *PTGER4*, *TNC*, and *WNK1* were associated with high enrichment binding of ALKBH5 in neutrophils (Fig. 6e). Therefore, these five genes are m^6^A targets, with their transcripts are strongly bound and directly modulated by ALKBH5.

m^6^A RNA modification modulates gene expression by affecting the alternative splicing, stability, and translation of mRNA.^10,11,39^ ALKBH5-mediated m^6^A demethylation has been demonstrated to promote or inhibit RNA decay of its different target transcripts under the same condition, respectively.^38,40,41^ There is only one isoform expression pattern of *Cxcr2*, *Nlrp12*, and *Tnc* transcripts. Besides, RNA-seq analysis indicated no significant difference in the alternative splicing pattern of *Ptger4* and *Wnk1* transcripts between WT and *Alkbh5*-deficient neutrophils (Supplementary Fig. 8a). RNA decay assays showed that the mRNA stability of *CXCR2* and *NLRP12* were significantly decreased in *ALKBH5*-deficient dHL-60 human neutrophils after transcription inhibition (Fig. 6f). Besides, loss of ALKBH5 markedly inhibited the mRNA decay of *PTGER4*, *TNC*, and *WNK1* in dHL-60 neutrophils treated with actinomycin-D for different hours (Fig. 6g).

Taken together, ALKBH5 specifically removes m^6^A methylation on its target mRNAs to regulate their RNA decay, so as to directly alter the mRNA expression and consequently modulate protein expression of a class of neutrophil-intrinsic and migration-related molecules (e.g. *Cxcr2*, *Nlrp12*, *Ptger4*, *Tnc*, and *Wnk1*) for neutrophil migration in antibacterial innate defense.

## Discussion

The previously undescribed role of m^6^A demethylase ALKBH5 is identified here in promoting antibacterial innate defense through intrinsic motivation of neutrophil migration. ALKBH5 empowers neutrophils to self-amplify their migration by: upregulation of neutrophil migration-promoting receptors that response to extracellular signals such as chemokines, whereas downregulation of neutrophil migration-suppressive molecules, through m^6^A demethylation-mediated changes in RNA decay. This intrinsically epigenetic mechanism enables neutrophil accumulation in the site of infection, and crucially, promotes effective bacterial clearance and hence prevents excess inflammatory responses.

Although a variety of extrinsic or intracellular molecules have been implicated in orchestrating the fates and behaviors of immune cells, it is largely unexplored whether immune cell migration might be intrinsically regulated by m^6^A RNA modification inside and its enzymes. We previously found that m^6^A modification is involved in CCR7-mediated DC migration by degradation of lnc-Dpf3,^42,43^ inspiring us to understand whether neutrophils can be well equipped with strong migration ability via m^6^A modification. During neuroinflammation, T cell-specific ALKBH5 ablation has been shown to diminish neutrophil recruitment into the central nervous system of mice with experimental autoimmune encephalomyelitis by decreasing *Cxcl2* mRNA stability in CD4^+^ T cells.^16^ Our findings in this study reveal previously unknown role of ALKBH5 and its m^6^A demethylation in neutrophil migration by endowing neutrophils with intrinsic ability to response to extrinsic chemokine signals and to migrate into infection sites. Indeed, *Alkbh5*-deficient neutrophils, which purified from bone marrow of mice in the steady state, display a significant defective migration ability toward CXCL2 in vitro. Moreover, deletion of ALKBH5 directly leads to alterations in RNA decay and consequent expression levels of neutrophil migration-related molecules in dHL-60 cells that infected with *E.coli* in vitro. These results together illustrate that nucleus located ALKBH5 intrinsically empowers neutrophil migration, providing new insights to the epigenetic mechanism for neutrophil migration in antibacterial innate defense.

As the major kind of effector cells in antibacterial defense, neutrophils might affect the recruitment or function of other immune cells including macrophages. The interplay between neutrophils and macrophages plays a central role in host innate defense against bacterial infection and sepsis.^44^ For instance, peritoneal neutrophil-derived CCL2 is critical for macrophage recruitment to local infection site during sepsis.^27^ In turn, abnormal levels of cytokines or chemokines produced by macrophages in inflamed sites can alter neutrophil recruitment.^45^ m^6^A RNA modification mediated by methyltransferase METTL3 has been identified as a positive regulator of macrophage activation through inducing the degradation of TLR signaling inhibitor *Irakm* transcripts, thus contributing to increased antibacterial activity of macrophages against *S. typhimurium* infection.^46^ Our results showed an indirect effect of ALKBH5 on promoting macrophage recruitment by increasing peritoneal CCL2 levels. In addition, RNA-seq data also indicated the down-regulated expression of some other chemokines and cytokines such as colony stimulating factor 1 (*Csf1*, also known as M-CSF) in *Alkbh5-*deficient neutrophils, which also likely explains the decreased macrophages given that neutrophil-derived CSF1 controls recruitment of these cells to inflamed site.^47^ It is worth investigating whether ALKBH5 directly affects macrophage migration or innate function during bacterial infection in the future.

Downregulated CXCR2 expression on circulating neutrophils during sepsis or bacterial infection has been demonstrated previously and is correlated with impaired neutrophil chemotaxis into infectious sites.^23,48^ Previous study shows that lower expression of CXCR2 on neutrophils lead to reduced neutrophil influx to the peritoneal cavity of CLP mice, accompanied by increased bacteria load and higher inflammatory cytokines in peritoneal cavity and blood,^2,49^ which are in agreement with our observations in *Alkbh5*-deficient CLP mice when compared to WT CLP mice. The expression of CXCR2 on neutrophils has been reported that can be down-regulated by: TLR4 activation-triggered expression of G protein-coupled receptor kinase-2 (GRK2), a serine-threonine protein kinase that causes CXCR2 be phosphorylated so as to induce CXCR2 internalization;^2^ or sepsis-induced decline in the level of acetylation of histone H3, an activation mark, at the CXCR2 promoter in neutrophils.^49^ Besides, extracellular mediators such as iNOS-derived NO also reduce CXCR2 expression in severe sepsis.^48^ Here we found that bacterial infection-induced downregulation of ALKBH5 might be responsible for decreased CXCR2 on neutrophils, and mechanistically, ALKBH5 promotes CXCR2 expression by removing m^6^A methylation on its RNA thus to prevent *CXCR2* mRNA degradation. In addition to the previous findings that focused mainly on the regulation of CXCR2 expression via post-translational modification or at gene transcription level, our present study reveals a previously unrecognized epigenetic mechanism, that is m^6^A RNA modification-mediated post-transcriptional regulation, in modulating the mRNA stability and expression of CXCR2 as well as other key players involved in neutrophil migration.

During pathogen-host interaction, the pathogens have developed strategies to escape from the host innate defense by disabling innate cell function or utilizing the negative regulatory mechanism of immune response, for instance, the epigenetic dysregulation caused by ALKBH5 downregulation, as observed in this study. One question remains regarding the mechanisms of bacterial infection-induced suppression of ALKBH5 in neutrophils. Bacterial infection-induced activation of TLR2 and TLR4 in neutrophils have been demonstrated to impair neutrophil migration.^2,23^ In epithelial cells, ALKBH5 is decreased in response to infection by *Streptococcus suis* and *Cryptosporidium parvum*, with the involvement of TLR/MYD88/NF-кB signaling activation.^50^ Future study is needed to reveal the mechanisms underlying downregulation of ALKBH5 during bacterial infection, for example, to clarify whether it is due to the activation of TLRs signaling or even reprogramming metabolic status, which will contribute to develop corresponding intervention strategies for bacterial infections.

Besides, ALKBH5 might enhance neutrophil expansion through regulating other biological process. It has been proposed that neutrophils have phenotypic heterogeneity and functional plasticity in tumor, ranging from pro-inflammatory, anti-tumor ‘N1’ neutrophils to anti-inflammatory, pro-tumor ‘N2’ neutrophils.^51^ Does a similar plasticity of neutrophils exist at sites of bacterial infection and under control of ALKBH5? Single-cell transcriptome profiling reveals that bacterial infection reprograms the structure of the neutrophil population and the dynamic transition between each subpopulation.^52^ Abdominal sepsis-induced emergency myelopoiesis, an important source of neutrophils, is reported to be promoted by Tet2-mediated mRNA oxidation, another kind of RNA modification.^18^ Our RNA-seq data imply that deletion of ALKBH5 also affects the expression of other genes involved in regulation of neutrophil behavior. For instance, *Alkbh5*-deficient neutrophils display decreased levels of granulopoiesis marker *Csf3r* (G-CSF receptor), and reduced expression of cell adhesion molecules including *Sell* (also known as L-selectin or CD62L), *selp* (also known as P-selectin or CD62P), and *Selplg* (selectin P ligand). The possible roles of ALKBH5 in distinct neutrophil subpopulations, emergency granulopoiesis, and neutrophil adhesion need further investigation.

Failure of neutrophil migration and lack of functional neutrophils in the infection site have been widely observed in sepsis patients and bacterial infections, which are associated with increased mortality and higher bacterial burden.^53–56^ How to reverse the suppressed state of neutrophil migration to restore and even improve antibacterial host defense is one of the most challenging issues for the treatment of severe bacterial infections or sepsis. Consisting with the decreased ALKBH5 level observed in severe sepsis patients, our findings show that ALKBH5 is down-expressed in primary human neutrophils during bacterial infection or in neutrophils from sepsis mice, and deficiency of ALKBH5 impairs neutrophil migration and antibacterial innate defense. Therefore, activation or upregulation of the ALKBH5-m^6^A demethylation axis, an intrinsic mechanism for driving efficient neutrophil migration, may be a potentially promising approach to the treatment of sepsis and other bacterial infectious diseases.

## Materials and methods

### Mice and animal models

C57BL/6 mice were from Beijing Vital River Laboratory (Beijing, China). *Alkbh5*-deficient mice (*Alkbh5*^–/–^) on a C57BL/6 background were obtained as before.^15,20^ Genotyping of the offspring mice by using wild-type (WT) primers: F1, 5ʹ- CGATCCGTGGTAAATCTG-3ʹ, R1, 5ʹ-TAAGTAAGTGCCTGAATGG-3ʹ; *Alkbh5*^–/–^ primers: F2, 5ʹ-AATCTGACGGAGTATCAAAGACTGGAAAAGG-3ʹ, R2, 5ʹ-AAGGAGACCACATTCATAGAACTCGAACTCC-3ʹ. All mice were bred and maintained under specific-pathogen-free conditions and 6–10-week-old littermate mice were used. All mouse experiments were performed according to the National Institutes of Health Guide for the Care and Use of Laboratory Animals, with the approval of the Animals Care and Use Committees of the Institute of Laboratory Animal Sciences of Chinese Academy of Medical Sciences (ACUC-A01-2020-004).

#### Cecal ligation and puncture

In all, 6–10-week-old mice were used in this study. The rodent model of sepsis was performed as previously described.^18,21^ All experiments included age-matched controls. In brief, the peritoneal cavity was opened after the mouse was anesthetized, and the cecum was exteriorized and ligated at different points distal of the ileo-cecal valve using a nonabsorbable 7-0 suture. To induce mid-grade sepsis (mild CLP), ~50% of the cecum was ligated. To induce high-grade sepsis (lethal), ~75% of the cecum was ligated. Only experiments testing survival used high-grade sepsis. The distal end of the cecum was then perforated using a 21 G needle, and a small drop of feces was extruded through the puncture. The cecum was relocated into the peritoneal cavity and the peritoneum was closed. Sham-operated animals that underwent identical laparotomy but without cecal puncture were used as controls.

### Cell isolation and culture

#### Neutrophil isolation

Mouse neutrophils were isolated from peritoneal cavity, peripheral blood, or bone marrow by Percoll density gradient as previously described.^23^ Human primary neutrophils were isolated from the peripheral blood of healthy human donors using gradient separation as previously described.^57^ Cells were resuspended in RPMI-1640 supplemented with 10% (v/v) fetal bovine serum (FBS, Gibco) for subsequent experiments or in 1× PBS for flow cytometry analysis.

Mouse bone marrow-derived macrophages (BMDMs) were prepared by culturing in endotoxin-free DMEM medium with 10% (v/v) FBS (Gibco) and recombinant mouse M-CSF (R&D Systems) and were treated as indicated on day 6.

HL-60 and RAW 264.7 cell lines were obtained from American Type Culture Collection (ATCC) and cultured as required. In detail, HL-60 cells were cultured in RPMI-1640 (w/o Hepes) medium supplemented with 10% (v/v) FBS (Gibco) and penicillin/streptomycin at 37 °C, 5% CO_2_. For neutrophil differentiation (dHL-60), the medium was supplemented with 1 μM retinoic acid (ATRA, Sigma) for HL-60 cells for 4 days.

### Ethics approval and consent to participate

The peripheral blood samples from healthy donors were collected in this study. Using of human subjects in this study was approved by The INSTITUTIONAL REVIEW BOARD of Institute of Basic Medical Sciences, Chinese Academy of Medical Sciences (Project No: 083-2022). The written informed consents were obtained from all participants before the study.

### Bacterial culture and infection

*Escherichia coli* (*E.coli*, JM109 strain, B528410-0001) was purchased from Sanger Biotech and grown in LB medium on a shaker at 37 °C overnight. For bacterial infection, dHL-60 cells, human primary neutrophils, or mouse neutrophils were infected with 1 × 10^6^ CFUs of *E.coli* (JM109 strain, 1:1 ratio) for indicated time.

### RNA extraction and quantitative RT-PCR

Total RNA was extracted with TRIzol reagent (Invitrogen) or RNAfast200 kit (FASTAGEN). In total, 1 μg acquired RNA was reversely transcribed into cDNA using ReverTra Ace qPCR RT Master Mix with gDNA Remover (FSQ-301, TOYOBO) according to the manufacturer’s instructions, then followed by real-time PCR analysis with SYBR Green Realtime PCR Master Mix (QPK-201, TOYOBO). Products were measured by QuantStudio 7 Flex (Thermo Fisher Scientific). The relative RNA expression level was normalized to *Gapdh* or *GAPDH* according to the ^△△^C_t_ calculation method. Primer sequences used for targets are shown in Supplementary Table 2.

### Western blot

The immunoblot analysis was performed as described previously.^15^ Briefly, cells were lysed with RIPA buffer (20–188, Millipore) supplemented with protease inhibitor cocktail and phosphatase inhibitor cocktail (Thermo Fisher Scientific). For the separation of nuclear and cytoplasmic proteins, cells were firstly lysed with cytoplasmic lysis buffer (Tris 10 mM, NaCl 10 mM, MgCl2 3 mM, Nonidet P-40 0.1%) supplemented with protease inhibitor cocktail and phosphatase inhibitor cocktail (Thermo Fisher Scientific) for 3 min, and the supernatant was collected for the detection of cytoplasmic proteins. After three washes with cytoplasmic lysis buffer, the nuclei were lysed with RIPA buffer. Protein concentrations were measured with BCA protein assay kit (Thermo Fisher Scientific). ALKBH5 (HPA007196) and METTL14 (HPA038002) antibodies were from Sigma. YTHDF1 (17479-1-AP) and PTGER4 (66921-1-Ig) antibodies were from Proteintech. METTL3 (ab195352), FTO (ab92821), and Tenascin C (ab108930) antibodies were from Abcam. WNK1 (MA5-35466) and NLRP12 (PA5-89879) antibodies were from Invitrogen. GAPDH (M171-3) and YTHDF2 (RN123PW) antibodies were from MBL. Lamin A/C (4777 S) antibody was from CST. Goat anti-rabbit IgG-HRP (ZB-2301) and goat anti-mouse IgG-HRP (ZB-2305) antibodies were from ZSGB-BIO.

### m^6^A dot blot

Total RNA was extracted with TRIzol reagent (Invitrogen) or RNAfast200 (FASTAGEN). Poly(A) + mRNA was isolated using the Dynabeads mRNA Purification Kit (61006, Invitrogen) according to the manufacturer’s instructions. RNA samples were quantified by 2200 Tape Station (Agilent). Equal amounts mRNA was denatured at 65 °C for 15 min followed by chilling on ice immediately and mixed at 3:2 ratio with glyoxal loading dye (Ambion). mRNA was dropped directly onto the Hybond-N + membrane (GE Healthcare) and performed UV crosslinking. The membranes were washed with 0.1% PBST (0.1% Tween-20 in 1 × PBS, pH 7.4) and blocked with 5% non-fat milk in 0.1% PBST (Blocking buffer). Then, the anti-m^6^A antibody (202003, Synaptic Systems) was diluted 1:500 in blocking buffer and incubated with the membranes for 2 h at room temperature with gentle shaking. The membranes were washed extensively and incubated with goat anti-Rabbit IgG-HRP (1:1,000 dilution, ZB-2301, ZSGB-BIO) for 2 h at room temperature. After extensive wash, the membranes were developed by enhanced chemiluminescence with Hyperfilm ECL (GE Healthcare). Equal RNA loading was verified by methylene blue (MB) staining.

### Bacterial counts

We determined bacterial counts by colony-forming unit (CFU) assay as described previously.^2^ Briefly, whole blood, peritoneal lavage fluid, and indicated organs were harvested from mice using standard techniques. Then, all samples were diluted by 1 × PBS and ultrasonic grinding, followed serially diluted and plated on LB agar dishes. Then incubated the dishes at 37 °C. The number of bacterial colonies was assessed 24 h later.

### ELISA

The concentrations of IL-6, IL-1β, CXCL2, CXCL1 or CCL2 in the peritoneal lavage fluid, plasma, or bone marrow of mice were determined with ELISA kits (R&D Systems, M6000B, MLB00C, MM200, MKC00B, MJE00B) according to the manufacturer’s instructions.

### Flow cytometry

Single-cells suspensions were obtained from peritoneal lavage fluid, peripheral blood, and bone marrow of 6 to 10-week-old *Alkbh5*^–/–^ mice and WT littermates, then were labeled with fluorescently labeled antibodies as described previously^15^ and filtered through 40-μm cell strainers. All the samples were analyzed on LSRFortessa (BD Biosciences) and analyzed with FlowJo (TreeStar). Antibodies that used for staining cells are as following: Mouse: PE-Cy5 anti-mouse CD45 (BD Pharmingen), APC or PE-Cy7 anti-mouse CD11b (BD Biosciences), FITC anti-mouse Ly6G (BD Biosciences), PerCP or PE-Cy7 anti-mouse F4/80 (Biolegend), APC anti-mouse CXCR2 (Biolegend). Human: PE anti-human CXCR2 (Biolegend). Cell apoptosis: APC Annexin V (Biolegend), PerCP 7-AAD (Biolegend). Cells were defined as: mouse neutrophils (Ly6G^+^ CD11b^+^), mouse macrophages (F4/80^+^ CD11b^+^), and apoptotic neutrophils (Ly6G^+^ CD11b^+^ Annexin V^+^).

### Transwell migration assay

In vitro migration assay was performed as previously described.^2^

#### For neutrophil migration assay

Neutrophils isolated from bone marrow of mice (1 × 10^6^ cells/ml) were incubated with indicated *E.coli* strains for 2 h. Then 200 μl neutrophils (1 × 10^6^ cells/ml) were allowed to migrate toward CXCL2 (30 ng/ml, R&D Systems) in 500 μl medium or medium alone at 37 °C with 5% CO2, in 24-well microchamber using 3-μm-pore polycarbonate Transwell plates (Corning). After 2 h, cells that migrated through the membrane were stained with FITC anti-mouse Ly6G (BD Biosciences) or Trypan Blue (Countstar) and counted by Automated Cell Counter (Countstar).

#### For macrophage migration assay

BMDMs or RAW 264.7 cells were seeded into the upper chambers of 8-μm-pore filter plates (Costar) with an approximate number of 4 × 10^4^ cells. Recombinant CCL2 chemokine (100 ng/ml, MCE) in 500 μl DMEM medium was added into the lower chamber. Plates were incubated in an incubator with the constant temperature of 37 °C and 5% CO_2_. Cells passed through membrane were harvested through a carefully removal of cells on the upper side by using wet cotton swabs. Filters were immersed in 4% paraformaldehyde (Solarbio) for half an hour, and then were stained with 0.1% crystal violet for 30 min. The cells on the filters were counted under a microscope after washing for three times.

### Neutrophil polarization assay

The polarization assay was performed as previously described.^3^ Briefly, neutrophils obtained from bone marrow of mice in the steady state were resuspended at a density of 1 × 10^6^ cells/ml in RPMI-1640 supplemented with 10% (v/v) FBS. Cells were incubated with 1 × 10^6^ CFUs/ml of *E.coli* (JM109 strain) for 2 h and then stimulated with CXCL2 (30 ng/ml, R&D Systems) for 1 h. Images were captured using a 40 × objective on an Evos FL Auto 2 microscope (Thermo Fisher Scientific). The percentage of neutrophils extending pseudopods or ruffling was calculated from fields captured at the indicated time points after chemoattractant stimulation.

### In vitro phagocytosis assay

In vitro phagocytosis assay was performed as previously described.^3,45^ Briefly, pHrodo Deep Red *E.coli* bioparticles (Invitrogen) were reconstituted in HBSS and opsonized with 12.5% mouse serum at 37 °C for 30 min. Bone marrow neutrophils from normal mice were infected with serum-opsonized pHrodo Deep Red *E.coli* bioparticles (1:1 ratio), which fluoresce brightly red only in low pH of phagosomes. After incubated at 37 °C for 2 h, cells were washed and resuspended in ice-cold HBSS. The internalized bioparticles were detected by FACS analysis. Phagocytosis efficiency (Phagocytosis index) was expressed as MFI of the internalized pHrodo Deep Red *E.coli* bioparticles per neutrophil.

### In vitro bacterial killing assay

In vitro bacterial killing assay was performed as previously described.^3,45^ Neutrophils were isolated from the bone marrow of mice in the steady state, and then were separated by centrifugation over a three-layer Percoll gradient. Live *E.coli* particles (JM109 strain, 1:1 ratio) were opsonized with mouse serum at 37 °C for 30 min and then incubated with neutrophils in HBSS (without mouse serum) at 37 °C for 2 h. Samples were then serially diluted and spread on LB agar plates and incubated at 37 °C. The number of live *E.coli* particles in each sample was determined after overnight incubation at 37 °C.

### RNA-seq

Total RNA was isolated from *Alkbh5*^–/–^ and WT peritoneal neutrophils with TRIzol reagent (Invitrogen) and then subjected to Poly(A) + mRNA purification via Dynabeads mRNA Purification Kit (61006, Invitrogen) according to the manufacturer’s instructions. RNA samples were quantified by 2200 Tape Station (Agilent). The RNA libraries were prepared with NEBNext Ultra II Directional RNA Library Prep Kit for Illumina (NEB) according to the manufacturer’s instructions. Four independent biological replicates were performed for RNA-seq.

### m^6^A specific methylated RNA immunoprecipitation combined with high-throughput sequencing (m^6^A-seq)

m^6^A-seq was as performed as our previous protocol.^15^ In detail, about 500 μg total RNA from cells were extracted with TRIzol reagent (Invitrogen) then subjected to Poly(A) + mRNA purification via Dynabeads mRNA Purification Kit (61006, Invitrogen) following the manufacturer’s instructions. Then RNA fragmentation, immunoprecipitated of m^6^A-containing RNA fragments, and library preparation were performed. Briefly, purified poly(A) + mRNA was fragmented into ~ 100 nt and incubated with anti-m^6^A antibody (202003, Synaptic System) for 2 h at 4 °C, then were immunoprecipitated by incubation with Protein A/G beads (Thermo Fisher Scientific) for 2 h at 4 °C. Captured RNA was competitively eluted by m^6^A nucleotide and purified by ethanol precipitation. The input mRNA sample (input) and purified mRNA sample (IP) were used for library construction by TruSeq Stranded mRNA Library Preparation kit (Illumina) and were quantified by BioAnalyzer High Sensitivity DNA chip according to the manufacturer’s instructions. Two independent biological replicates were performed for m^6^A-seq.

### Analysis of high-throughput sequencing data

#### General processing

All samples were sequenced on Illumina NovaSeq 6000 with paired-end. Samples were sequenced together in one flow cell in two lanes, and the reads from two lanes of each sample were combined for analysis. After removing adapters and low-quality bases, the Fastq files were aligned to the reference genome (mm10 or hg38) using Hisat2. Reads mapped to tRNA and rRNA were removed and each sample obtained ~25 million useful reads for the following analysis.

#### RNA-seq and gene expression analysis

Stringtie (v2.1.4) was used to calculate the TPM of each gene to represent their mRNA expression level. The differential genes were identified by a negative binomial model using the DEseq2 package, combining information from all replicates. The significantly differentially expressed genes have to meet all following criteria: *P* value ≤ 0.05, log_2_ (fold-change) ≥1 or log_2_ (fold-change) ≤−1. The Gene Ontology biological processes enrichment analysis of differentially expressed genes were conducted by R package ClusterProfiler (v3.14.3).

#### m^6^A-seq analysis

On the basis of our m^6^A-seq data (GSE127732), the m^6^A peak calling was processed as our previously described.^15^ The longest isoform of each gene was scanned using a 100-bp sliding window with 10-bp steps. We excluded windows with read counts less than 1/20 of the top window in both the input and m^6^A-IP sample to reduce bias from potentially inaccurate gene structure annotation and the arbitrary use of the longest isoform. Sequence motifs on m^6^A peaks and *P* value were identified by HOMER.

### Generation of ALKBH5 knockout cell line

ALKBH5 knockout HL-60 cell line was constructed by using the CRISPR-Cas9 gene-editing system. Single-guide RNA (sgRNA) targeting sequences (5ʹ-GGCCAAGCGCAAGTATCAGGAGG-3ʹ and 5ʹ-GCTGGTGATCCAAAAGCTGGTGG-3ʹ) were designed using the MIT online tool (http://crispr.mit.edu/), then synthesized and inserted into the pGL3-U6-sgRNA expression vector (Addgene). For generating ALKBH5-knockout cells, HL-60 cells were transfected with human ALKBH5-targeting, control sgRNA expression vectors and Cas9 expression plasmids (Addgene) by electroporation using SF Cell Line 4D-NucleofectorTM X Kit L and Lonza Nucleofector™4D (Lonza). Then, HL-60 cells were selected with 0.0875 μg/ml puromycin (Invivogen) and 1.5 μg/ml blasticidin (Invivogen) in culture medium for 1 week. Single-cell colonies were picked and the knockout efficiency was determined by genomic DNA sequencing and Western blot at DNA and protein expression levels.

### RNA immunoprecipitation-qPCR

RIP-qPCR assay was performed as previously described.^41^ Briefly, neutrophils (~3 × 10^7^ cells for each sample) were harvested and lysed in IP lysis buffer (Thermo Scientific) and then incubated with 10 μg anti-ALKBH5 antibody (Sigma) or 10 μg control anti-IgG antibody (Millipore) at 4 °C overnight. Then, the cell lysates were mixed with protein A/G beads (Thermo Scientific) at 4 °C for 2 h. The beads were washed 6 times using IP lysis buffer and then resuspended in proteinase K to incubate at 56 °C for 1 h. The immunoprecipitated and input RNAs were isolated using the TRIzol reagent for further RT-qPCR analysis.

### RNA decay assay

*ALKBH5*^-/-^ and WT dHL-60 cells were seeded at a density of 1 × 10^6^ cells/ml in 12-well plates, actinomycin D (A1410, Sigma) was added to the cell medium to block de novo RNA synthesis at a final concentration of 5 μg/ml. After incubation for indicated time points, cells were collected and RNA samples were extracted for qRT-PCR to determine the mRNA levels of indicated genes. The data were normalized to the *t* = 0 time point.

### Statistical analysis

Data were expressed as mean ± SEM. *P* values were calculated using two-tailed unpaired Student’s *t*-test for pairwise comparison of variables, or Kaplan-Meier for survival curves. All general statistical analyses were used a confidence interval of 95%. Sample sizes were determined on the basis of previous experiments using similar methodologies and were detailed in each figure legend. Data shown are representative of at least three independent experiments, including blots. For in vivo studies, mice were randomly assigned to treatment groups. All stated replicates are biological replicates.

## Supplementary information

Supplementary Materials

## Data Availability

All data are available in the manuscript or the supplementary materials. RNA-seq and m^6^A-seq raw data have been deposited in the NCBI Gene Expression Omnibus database under accession numbers GSE198316, GSE201060, and GSE127732, respectively.
