# Supplementary material for: m6A demethylase ALKBH5 is required for antibacterial innate defense by intrinsic motivation of neutrophil migration
Source: Signal Transduct Target Ther. 2022 Jun 29;7:194. doi: 10.1038/s41392-022-01020-z (PMC9240034; doi:10.1038/s41392-022-01020-z)
Supplement: Supplementary file 1 — Supplementary Materials [file 41392_2022_1020_MOESM1_ESM.pdf]

Supplementary Materials for

m^{6}A demethylase ALKBH5 is required for antibacterial innate defense by intrinsic motivation of neutrophil migration

Yang Liu[1,2,*], Renjie Song[1], Lu Zhao[1], Zhike Lu[3], Yini Li[3], Xinyi Zhan[1], Fengjiao Lu[1], Jiang Yang[1], Yamei Niu[4], Xuetao Cao[1,2,*]

Correspondence to: Yang Liu (yliu@immunol.org) or Xuetao Cao (caoxt@immunol.org)

This PDF file includes:

Materials and Methods

Figures S1 to S8

Tables S1 to S2

References

Materials and Methods

Mice and animal models

C57BL/6 mice were from Beijing Vital River Laboratory (Beijing, China). *Alkbh5*-deficient mice (*Alkbh5*$^{-/-}$) on a C57BL/6 background were obtained as before[1,2]. Genotyping of the offspring mice by using wild-type (WT) primers: F1, 5′- CGATCCGTGGTAAATCTG -3′, R1, 5′-TAAGTAAGTGCCTGAATGG-3′; *Alkbh5*$^{-/-}$ primers: F2, 5′-AATCTGACGGAGTATCAAAGACTGGAAAAGG-3′, R2, 5′-AAGGAGACCACATTCATAGAACTCGAACTCC-3′. All mice were bred and maintained under specific-pathogen-free conditions and 6 to 10-week-old littermate mice were used. All mouse experiments were performed according to the National Institutes of Health Guide for the Care and Use of Laboratory Animals, with the approval of the Animals Care and Use Committees of the Institute of Laboratory Animal Sciences of Chinese Academy of Medical Sciences (ACUC-A01-2020-004).

Cecal ligation and puncture (CLP): 6 to 10-week-old mice were used in this study. The rodent model of sepsis was performed as previously described[3,4]. All experiments included age-matched controls. In brief, the peritoneal cavity was opened after the mouse was anesthetized, and the cecum was exteriorized and ligated at different points distal of the ileo-cecal valve using a nonabsorbable 7-0 suture. To induce mid-grade sepsis (mild CLP), approximately 50% of the caecum was ligated. To induce high-grade sepsis (lethal), approximately 75% of the caecum was ligated. Only experiments testing survival used high-grade sepsis. The distal end of the cecum was then perforated using a 21 G needle, and a small drop of feces was extruded through the puncture. The cecum was relocated into the peritoneal cavity and the peritoneum was closed. Sham-operated animals that underwent identical laparotomy but without cecal puncture were used as controls.

Cell isolation and culture

Neutrophil isolation: Mouse neutrophils were isolated from peritoneal cavity, peripheral blood, or bone marrow by Percoll density gradient as previously described[5]. Human primary neutrophils were isolated from the peripheral blood of healthy human donors using gradient separation as previously described[6]. Cells were resuspended in RPMI-1640 supplemented with

10% (v/v) fetal bovine serum (FBS, Gibco) for subsequent experiments or in 1 × PBS for flow cytometry analysis.

Mouse bone marrow-derived macrophages (BMDMs) were prepared by culturing in endotoxin-free DMEM medium with 10% (v/v) FBS (Gibco) and recombinant mouse M-CSF (R&D Systems) and were treated as indicated on day 6.

HL-60 and RAW 264.7 cell lines were obtained from American Type Culture Collection (ATCC) and cultured as required. In detail, HL-60 cells were cultured in RPMI-1640 (w/o Hepes) medium supplemented with 10% (v/v) FBS (Gibco) and penicillin/streptomycin at 37°C, 5% CO2. For neutrophil differentiation (dHL-60), the medium was supplemented with 1 μM retinoic acid (ATRA, Sigma) for HL-60 cells for 4 days.

Ethics approval and consent to participate

The peripheral blood samples from healthy donors were collected in this study. Using of human subjects in this study was approved by The INSTITUTIONAL REVIEW BOARD of Institute of Basic Medical Sciences, Chinese Academy of Medical Sciences (Project No: 083-2022). The written informed consents were obtained from all participants before the study.

Bacterial culture and infection

Escherichia coli (*E.coli*, JM109 strain, B528410-0001) was purchased from Sanger Biotech and grown in LB medium on a shaker at 37°C overnight. For bacterial infection, dHL-60 cells, human primary neutrophils, or mouse neutrophils were infected with 1×10^6 CFUs of *E.coli* (JM109 strain, 1:1 ratio) for indicated time.

RNA extraction and quantitative RT-PCR

Total RNA was extracted with TRIzol reagent (Invitrogen) or RNAfast200 kit (FASTAGEN). 1 μg acquired RNA was reversely transcribed into cDNA using ReverTra Ace qPCR RT Master Mix with gDNA Remover (FSQ-301, TOYOBO) according to the manufacturer's instructions, then followed by real-time PCR analysis with SYBR Green Realtime PCR Master Mix (QPK-201, TOYOBO). Products were measured by QuantStudio 7 Flex (Thermo Fisher Scientific). The relative RNA expression level was normalized to *Gapdh* or

GAPDH according to the $^{\triangle\triangle}C_t$ calculation method. Primer sequences used for targets are shown in Supplementary Table. 2.

Western blot

The immunoblot analysis was performed as described previously[2]. Briefly, cells were lysed with RIPA buffer (20-188, Millipore) supplemented with protease inhibitor cocktail and phosphatase inhibitor cocktail (Thermo Fisher Scientific). For the separation of nuclear and cytoplasmic proteins, cells were firstly lysed with cytoplasmic lysis buffer (Tris 10mM, NaCl 10mM, MgCl2 3mM, Nonidet P-40 0.1%) supplemented with protease inhibitor cocktail and phosphatase inhibitor cocktail (Thermo Fisher Scientific) for 3 minutes, and the supernatant was collected for the detection of cytoplasmic proteins. After three washes with cytoplasmic lysis buffer, the nuclei were lysed with RIPA buffer. Protein concentrations were measured with BCA protein assay kit (Thermo Fisher Scientific). ALKBH5 (HPA007196) and METTL14 (HPA038002) antibodies were from Sigma. YTHDF1 (17479-1-AP) and PTGER4 (66921-1-Ig) antibodies were from Proteintech. METTL3 (ab195352), FTO (ab92821), and Tenascin C (ab108930) antibodies were from Abcam. WNK1 (MA5-35466) and NLRP12 (PA5-89879) antibodies were from Invitrogen. GAPDH (M171-3) and YTHDF2 (RN123PW) antibodies were from MBL. Lamin A/C (4777S) antibody was from CST. Goat anti-rabbit IgG-HRP (ZB-2301) and goat anti-mouse IgG-HRP (ZB-2305) antibodies were from ZSGB-BIO.

m^6A dot blot

Total RNA was extracted with TRIzol reagent (Invitrogen) or RNAfast200 (FASTAGEN). Poly(A)+ mRNA was isolated using the Dynabeads mRNA Purification Kit (61006, Invitrogen) according to the manufacturer's instructions. RNA samples were quantified by 2200 Tape Station (Agilent). Equal amounts mRNA was denatured at 65°C for 15 minutes followed by chilling on ice immediately and mixed at 3:2 ratio with glyoxal loading dye (Ambion). mRNA was dropped directly onto the Hybond-N+ membrane (GE Healthcare) and performed UV crosslinking. The membranes were washed with 0.1% PBST (0.1% Tween-20 in 1×PBS, pH 7.4) and blocked with 5% non-fat milk in 0.1% PBST (Blocking buffer). Then, the anti-m^6A antibody (202003, Synaptic Systems) was diluted 1:500 in blocking buffer and incubated with the membranes for 2 hours at room temperature with gentle shaking. The membranes were washed

extensively and incubated with goat anti-Rabbit IgG-HRP (1:1,000 dilution, ZB-2301, ZSGB-BIO) for 2 hours at room temperature. After extensive wash, the membranes were developed by enhanced chemiluminescence with Hyperfilm ECL (GE Healthcare). Equal RNA loading was verified by methylene blue (MB) staining.

Bacterial counts

We determined bacterial counts by colony-forming unit (CFU) assay as described previously[7]. Briefly, whole blood, peritoneal lavage fluid, and indicated organs were harvested from mice using standard techniques. Then, all samples were diluted by 1 × PBS and ultrasonic grinding, followed serially diluted and plated on LB agar dishes. Then incubated the dishes at 37ºC. The number of bacterial colonies was assessed 24 hours later.

ELISA

The concentrations of IL-6, IL-1β, CXCL2, CXCL1 or CCL2 in the peritoneal lavage fluid, plasma, or bone marrow of mice were determined with ELISA kits (R&D Systems, M6000B, MLB00C, MM200, MKC00B, MJE00B) according to the manufacturer's instructions.

Flow cytometry

Single-cells suspensions were obtained from peritoneal lavage fluid, peripheral blood, and bone marrow of 6 to 10-week-old *Alkbh5*$^{-/-}$ mice and WT littermates, then labeled with fluorescently labeled antibodies as described previously[2]. All the samples were analyzed on LSRFortessa (BD) and FlowJo. Antibodies that used for staining cells are as following: Mouse: PE-Cy5 anti-mouse CD45 (BD Pharmingen), APC or PE-Cy7 anti-mouse CD11b (BD Biosciences), FITC anti-mouse Ly6G (BD Biosciences), PerCP or PE-Cy7 anti-mouse F4/80 (Biolegend), APC anti-mouse CXCR2 (Biolegend). Human: PE anti-human CXCR2 (Biolegend). Cell apoptosis: APC Annexin V (Biolegend), PerCP 7-AAD (Biolegend). Cells were defined as: mouse neutrophils (Ly6G^{+} CD11b^{+}), mouse macrophages (F4/80^{+} CD11b^{+}), and apoptotic neutrophils (Ly6G^{+} CD11b^{+} Annexin V^{+}).

Transwell migration assay

In vitro migration assay was performed as previously described[7].

For neutrophil migration assay: Neutrophils isolated from bone marrow of mice (1×10^6 cells/ml) were incubated with indicated *E.coli* strains for 2 hours. Then 200 μl neutrophils (1×10^6 cells/ml) were allowed to migrate toward CXCL2 (30 ng/ml, R&D Systems) in 500 μl medium or medium alone at 37 °C with 5% CO2, in 24-well microchamber using 3-μm-pore polycarbonate Transwell plates (Corning). After 2 hours, cells that migrated through the membrane were stained with FITC anti-mouse Ly6G (BD Biosciences) or Trypan Blue (Countstar) and counted by Automated Cell Counter (Countstar).

For macrophage migration assay: BMDMs or RAW 264.7 cells were seeded into the upper chambers of 8-μm-pore filter plates (Costar) with an approximate number of 4×10^4 cells. Recombinant CCL2 chemokine (100 ng/ml, MCE) in 500 μl DMEM medium was added into the lower chamber. Plates were incubated in an incubator with the constant temperature of 37°C and 5% CO2. Cells passed through membrane were harvested through a carefully removal of cells on the upper side by using wet cotton swabs. Filters were immersed in 4% paraformaldehyde (Solarbio) for half an hour, and then were stained with 0.1% crystal violet for 30 minutes. The cells on the filters were counted under a microscope after washing for three times.

Neutrophil polarization assay

The polarization assay was performed as previously described[8]. Briefly, neutrophils obtained from bone marrow of mice in the steady state were resuspended at a density of 1×10^6 cells/ml in RPMI-1640 supplemented with 10% (v/v) FBS. Cells were incubated with 1×10^6 CFUs/ml of *E.coli* (JM109 strain) for 2 hours and then were stimulated with CXCL2 (30 ng/ml, R&D Systems) for 1 hour. Images were captured using a 40× objective on an Evos FL Auto 2 microscope (Thermo Fisher Scientific). The percentage of neutrophils extending pseudopods or ruffling was calculated from fields captured at the indicated time points after chemoattractant stimulation.

In vitro phagocytosis assay

In vitro phagocytosis assay was performed as previously described[8,9]. Briefly, pHrodo Deep Red *E.coli* bioparticles (Invitrogen) were reconstituted in HBSS and opsonized with 12.5% mouse serum at 37°C for 30 minutes. Bone marrow neutrophils from normal mice were infected with serum-opsonized pHrodo Deep Red *E.coli* bioparticles (1:1 ratio), which fluoresce brightly

red only in low pH of phagosomes. After incubated at 37°C for 2 hours, cells were washed and resuspended in ice-cold HBSS. The internalized bioparticles were detected by FACS analysis. Phagocytosis efficiency (Phagocytosis index) was expressed as MFI of the internalized pHrodo Deep Red *E.coli* bioparticles per neutrophil.

In vitro bacterial killing assay

In vitro bacterial killing assay was performed as previously described[8,9]. Neutrophils were isolated from the bone marrow of mice in the steady state, and then were separated by centrifugation over a three-layer Percoll gradient. Live *E.coli* particles (JM109 strain, 1:1 ratio) were opsonized with mouse serum at 37°C for 30 minutes and then incubated with neutrophils in HBSS (without mouse serum) at 37°C for 2 hours. Samples were then serially diluted and spread on LB agar plates and incubated at 37°C. The number of live *E.coli* particles in each sample was determined after overnight incubation at 37°C.

RNA-seq

Total RNA was isolated from *Alkbh5*$^{-/-}$ and WT peritoneal neutrophils with TRIzol reagent (Invitrogen) and then subjected to Poly(A)+ mRNA purification via Dynabeads mRNA Purification Kit (61006, Invitrogen) according to the manufacturer's instructions. RNA samples were quantified by 2200 Tape Station (Agilent). The RNA libraries were prepared with NEBNext Ultra II Directional RNA Library Prep Kit for Illumina (NEB) according to the manufacturer's instructions. Four independent biological replicates were performed for RNA-seq.

m^6A specific methylated RNA immunoprecipitation combined with high-throughput sequencing (m^6A-seq)

m^6A-seq was as performed as our previous protocol[2]. In detail, about 500 μg total RNA from cells were extracted with TRIzol reagent (Invitrogen) then subjected to Poly(A)+ mRNA purification via Dynabeads mRNA Purification Kit (61006, Invitrogen) following the manufacturer's instructions. Then RNA fragmentation, immunoprecipitated of m^6A-containing RNA fragments, and library preparation were performed. Briefly, purified poly(A)+ mRNA was fragmented into ~100 nt and incubated with anti-m^6A antibody (202003, Synaptic System) for 2 hours at 4°C, then were immunoprecipitated by incubation with Protein A/G beads (Thermo

Fisher Scientific) for 2 hours at 4°C. Captured RNA was competitively eluted by m^6A nucleotide and purified by ethanol precipitation. The input mRNA sample (input) and purified mRNA sample (IP) were used for library construction by TruSeq Stranded mRNA Library Preparation kit (Illumina) and were quantified by BioAnalyzer High Sensitivity DNA chip according to the manufacturer's instructions. Two independent biological replicates were performed for m^6A-seq.

Analysis of high-throughput sequencing data

General processing: All samples were sequenced on Illumina NovaSeq 6000 with paired-end. Samples were sequenced together in one flow cell in two lanes, and the reads from two lanes of each sample were combined for analysis. After removing adapters and low-quality bases, the Fastq files were aligned to the reference genome (mm10 or hg38) using Hisat2. Reads mapped to tRNA and rRNA were removed and each sample obtained ~ 25 million useful reads for the following analysis.

RNA-seq and gene expression analysis: Stringtie (v2.1.4) was used to calculate the TPM of each gene to represent their mRNA expression level. The differential genes were identified by a negative binomial model using the DEseq2 package, combining information from all replicates. The significantly differentially expressed genes have to meet all following criteria: P value $\leq$ 0.05, $\log_2$ (fold-change) $\geq$ 1 or $\log_2$ (fold-change) $\leq$ -1. The Gene Ontology biological processes enrichment analysis of differentially expressed genes were conducted by R package clusterProfiler (v3.8.1).

m^6A-seq analysis: On the basis of our m^6A-seq data (GSE127732), the m^6A peak calling was processed as our previously described[2]. The longest isoform of each gene was scanned using a 100-bp sliding window with 10-bp steps. We excluded windows with read counts less than 1/20 of the top window in both the input and m^6A-IP sample to reduce bias from potentially inaccurate gene structure annotation and the arbitrary use of the longest isoform. Sequence motifs on m^6A peaks and P value were identified by HOMER.

Generation of ALKBH5 knockout cell line

ALKBH5 knockout HL-60 cell line was constructed by using the CRISPR-Cas9 gene-editing system. Single-guide RNA (sgRNA) targeting sequences (5′-GGCCAAGCGCAAGTATCAGGAGG-3′ and 5′-GCTGGTGATCCAAAAGCTGGTGG-3′)

were designed using the MIT online tool (http://crispr.mit.edu/), then synthesized and inserted into the pGL3-U6-sgRNA expression vector (Addgene). For generating ALKBH5-knockout cells, HL-60 cells were transfected with human ALKBH5-targeting, control sgRNA expression vectors and Cas9 expression plasmids (Addgene) by electroporation using SF Cell Line 4D-NucleofectorTM X Kit L and Lonza Nucleofector™4D (Lonza). Then, HL-60 cells were selected with 0.0875 µg/ml puromycin (Invivogen) and 1.5 µg/ml blasticidin (Invivogen) in culture medium for 1 week. Single-cell colonies were picked and the knockout efficiency was determined by genomic DNA sequencing and Western blot at DNA and protein expression levels.

RNA immunoprecipitation (RIP)-qPCR

RIP-qPCR assay was performed as previously described[10]. Briefly, neutrophils (approximately 3×10^7 cells for each sample) were harvested and lysed in IP lysis buffer (Thermo Scientific) and then incubated with 10 µg anti-ALKBH5 antibody (Sigma) or 10 µg control anti-IgG antibody (Millipore) at 4°C overnight. Then, the cell lysates were mixed with protein A/G beads (Thermo Scientific) at 4°C for 2 hours. The beads were washed 6 times using IP lysis buffer and then resuspended in proteinase K to incubate at 56°C for 1 hour. The immunoprecipitated and input RNAs were isolated using the TRIzol reagent for further RT-qPCR analysis.

RNA decay assay

ALKBH5$^{-/-}$ and WT dHL-60 cells were seeded at a density of 1×10^6 cells/ml in 12-well plates, actinomycin D (A1410, Sigma) was added to the cell medium to block de novo RNA synthesis at a final concentration of 5 µg/ml. After incubation for indicated time points, cells were collected and RNA samples were extracted for qRT-PCR to determine the mRNA levels of indicated genes. The data were normalized to the $t = 0$ time point.

Statistical analysis

Data were expressed as mean ± SEM. *P* values were calculated using two-tailed unpaired Student's *t*-test for pairwise comparison of variables, or Kaplan-Meier for survival curves. All general statistical analyses were used a confidence interval of 95%. Sample sizes were determined on the basis of previous experiments using similar methodologies and were detailed

in each figure legend. Data shown are representative of at least three independent experiments, including blots. For *in vivo* studies, mice were randomly assigned to treatment groups. All stated replicates are biological replicates.

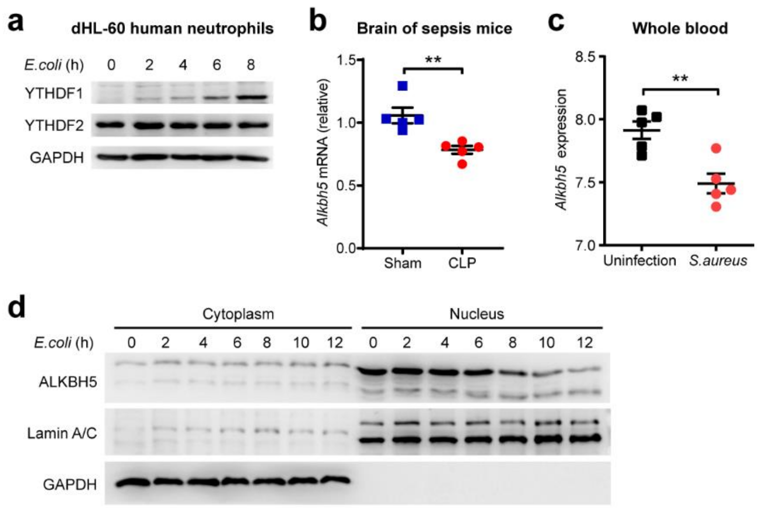

Figure. S1. ALKBH5 is down-expressed upon bacterial infection and mainly located in the nucleus of neutrophils.

a. Immunoblot analysis of protein levels of indicated m^{6}A readers in the differentiated HL-60 neutrophil-like cells (dHL-60) infected with *E.coli* for the indicated times. **b.** qRT-PCR analysis of *Alkbh5* mRNA expression in brain of mild CLP or sham- operated wild-type (WT) mice (*n*=5). qRT-PCR data were normalized to *Gapdh* expression. **c.** The *Alkbh5* expression in whole-blood from uninfected mice (*n*=5) or mice infected with *S. aureus* for 24h (*n*=5), based on the GSE38531 datasets. The expression values were showed as transformed count. *S. aureus*, *Staphylococcus aureus.* **d.** Immunoblot analysis of ALKBH5 in the cytoplasm and nucleus of dHL-60 cells infected with 1×10^5 CFUs of *E.coli* as indicated times. Lamin A/C and GAPDH were used as the nuclear and cytoplasmic controls, respectively. All data are mean ± SEM of biologically independent samples. Data are representative of 3 independent experiments with similar results [(**a**) and (**d**)]. $^{**}P < 0.01$. Two-tailed unpaired Student's *t* test (**b, c**).

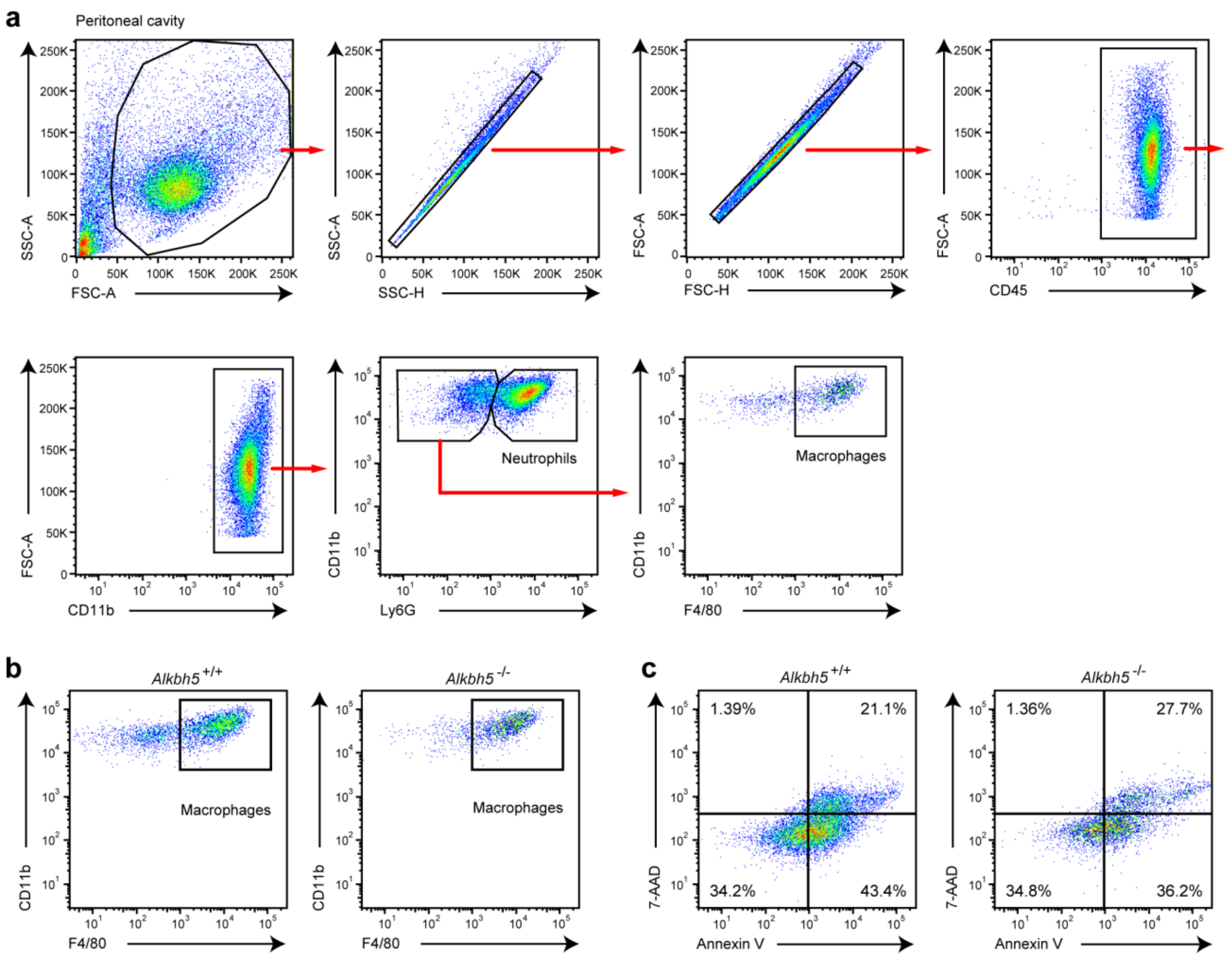

Figure. S2. FACS analysis of neutrophils and macrophages in the peritoneal cavity of sepsis mice.

a. Gating strategies for analyzing neutrophils and macrophages in the peritoneal cell population of CLP mice. Related to Fig.2. **b.** FACS analysis of macrophages in the peritoneal cavity of *Alkbh5*-deficient mice and WT littermates at 36h after mild CLP. Related to Fig.2d. **c.** FACS analysis of apoptotic neutrophils in the peritoneal cavity of mice as in (**b**). Related to Fig.2e. Data are representative of 3 to 10 independent experiments with similar results [(**a**) to (**c**)].

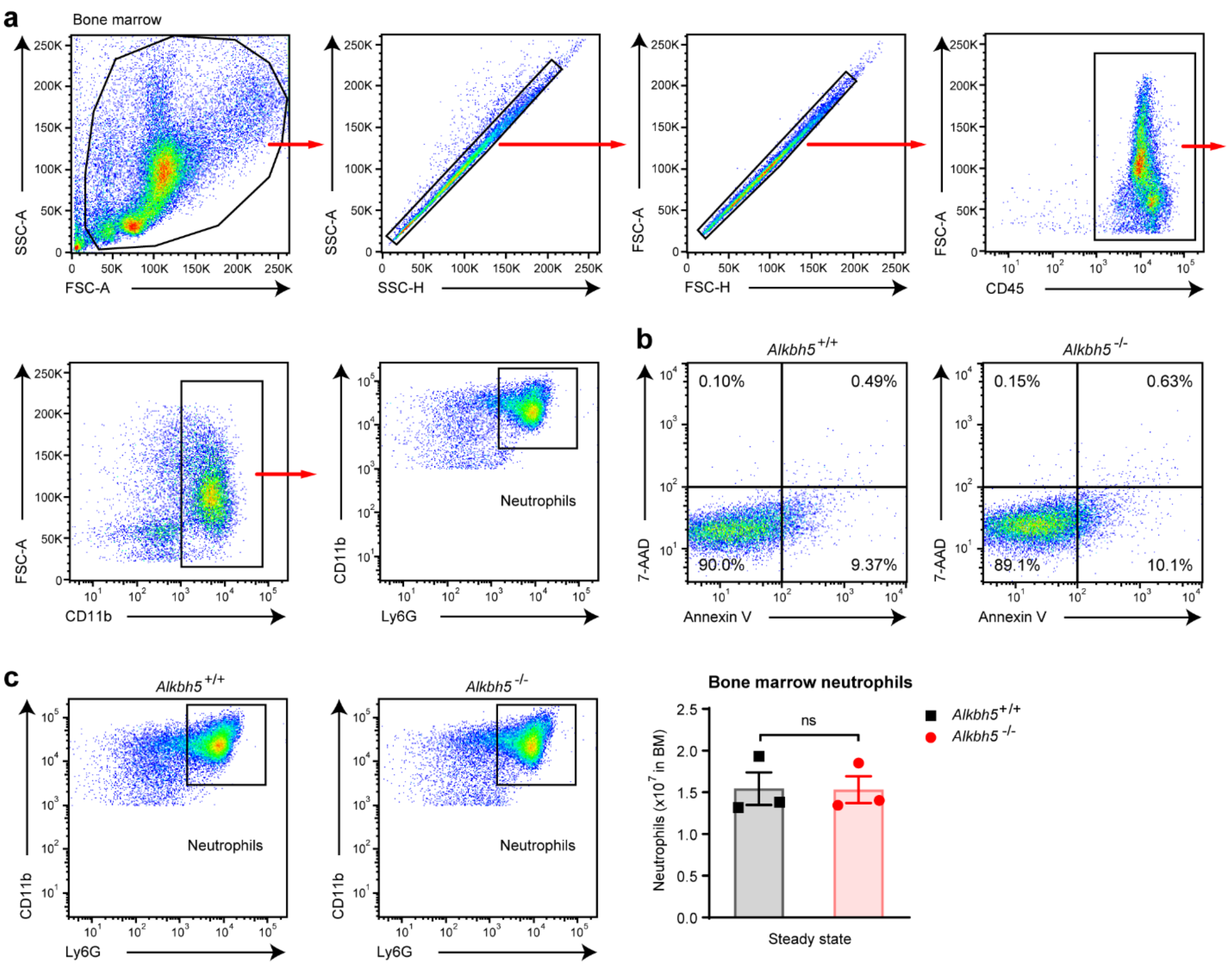

Figure. S3. ALKBH5 deficiency does not affect the number or apoptosis of bone marrow neutrophils in the steady state.

a. Gating strategies for analyzing neutrophils in bone marrow of mice. **b.** FACS analysis of the apoptosis of neutrophils in bone marrow of *Alkbh5*-deficient mice and WT littermates in the steady state. Related to Fig. 2f. **c.** FACS analysis of neutrophil number in bone marrow of *Alkbh5*-deficient mice and WT littermates in the steady state (*n*=3). BM, bone marrow. All data are mean ± SEM of biologically independent samples. Data are representative of 3 or 10 independent experiments with similar results (**a-c**). ns, not significant. Two-tailed unpaired Student's *t* test (**c**).

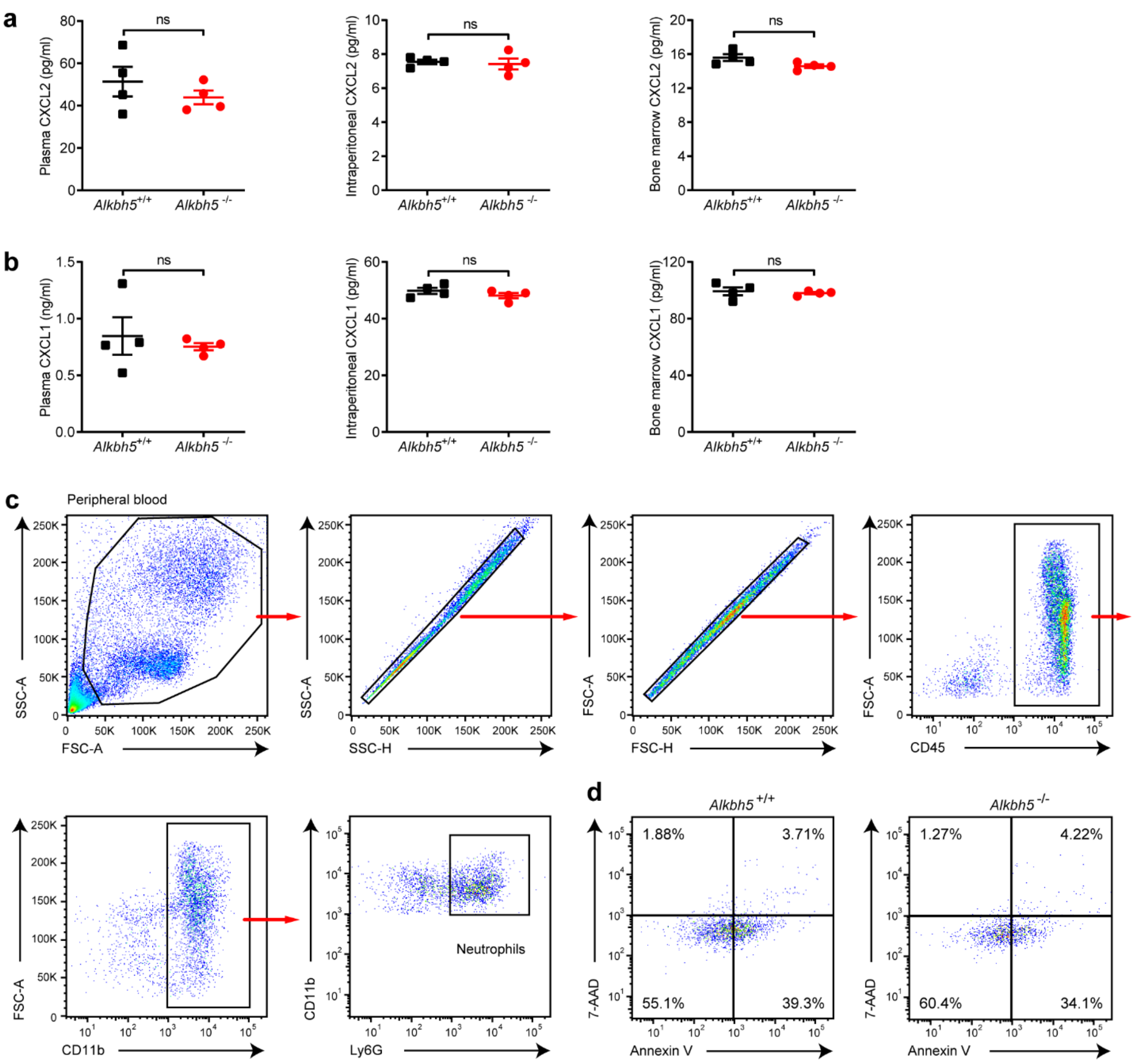

Figure. S4. *In vivo* deficiency of ALKBH5 does not alter chemokine levels in the steady state or neutrophil apoptosis during sepsis.

a-b. ELISA of CXCL2 (**a**) and CXCL1 (**b**) concentrations in the plasma, peritoneal cavity, or bone marrow of *Alkbh5*-deficient mice and WT littermates in the steady state (*n*=4). **c.** Gating strategies for analyzing neutrophils in peripheral blood of mice given mild CLP. **d.** FACS analysis of the apoptosis of neutrophils in peripheral blood of *Alkbh5*-deficient mice and WT littermates at 36h after mild CLP. Related to Fig. 3d. All data are mean ± SEM of biologically independent samples. Data are representative of 3 or 10 independent experiments with similar results (**c, d**). ns, not significant. Two-tailed unpaired Student's *t* test (**a, b**).

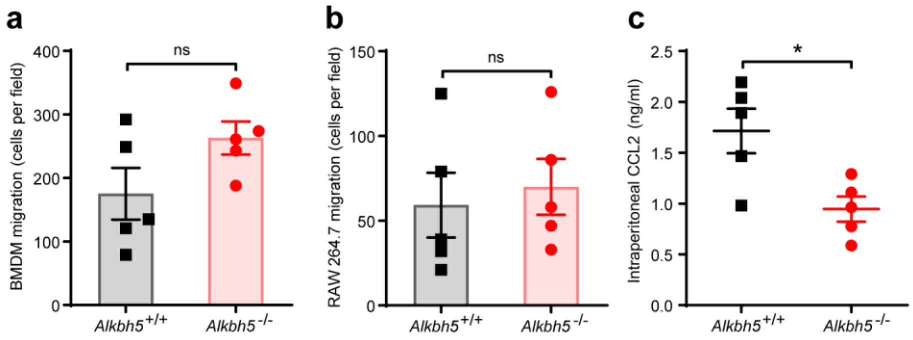

Figure. S5. ALKBH5 enhances peritoneal CCL2 production to induce effective macrophage recruitment during sepsis.

a. Transwell migration assay of bone marrow-derived macrophage (BMDM) from *Alkbh5*-deficient mice and WT littermates migration toward CCL2 (100 ng/ml) (n=5). **b.** Transwell migration assay of *Alkbh5*-deficient and WT RAW 264.7 macrophage migration toward CCL2 (100 ng/ml) (n=5). **c.** ELISA of CCL2 in the peritoneal cavity of *Alkbh5*-deficient mice and WT littermates at 36h after mild CLP (n=5). All data are mean ± SEM of biologically independent samples. *$P < 0.05$. ns, not significant. Two-tailed unpaired Student's t test [(**a**) to (**c**)].

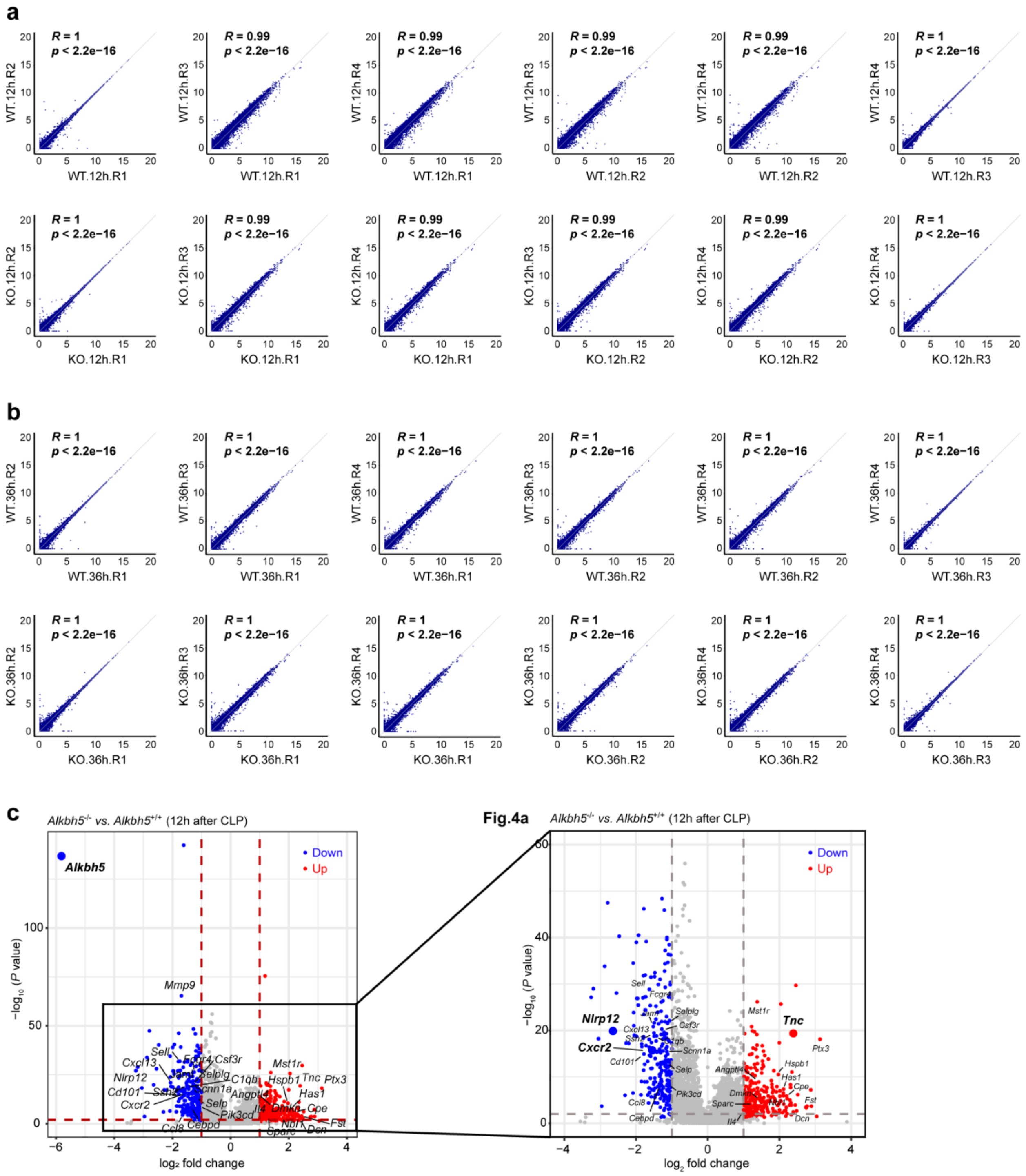

Figure. S6. ALKBH5 imprints migration-promoting transcriptome signatures in neutrophils in antibacterial defense.

a-b. Scatter plots showing a strong correlation among four biological replicates (R1 to R4) for 12h (**a**) and 36h (**b**) of RNA-seq. WT, wild-type; KO, *Alkbh5*-deficient. Pearson correlation

coefficient (*R*) and *P* value (*p*) were shown in the top left corner. **c.** Volcano plot of gene expression profiles in peritoneal neutrophils from *Alkbh5*-deficient mice (*Alkbh5*$^{-/-}$) and WT littermates (*Alkbh5*$^{+/+}$) at 12h after mild CLP. Fig.4a (right) was zoomed-in partial region indicated by the black box of complete volcano plot (left). Genes with significant changes in expression upon *Alkbh5* deficiency were colored by red for up-regulated genes and by blue for down-regulated genes. *Alkbh5* and its potential targets were labeled near bigger plots. Four independent biological replicates.

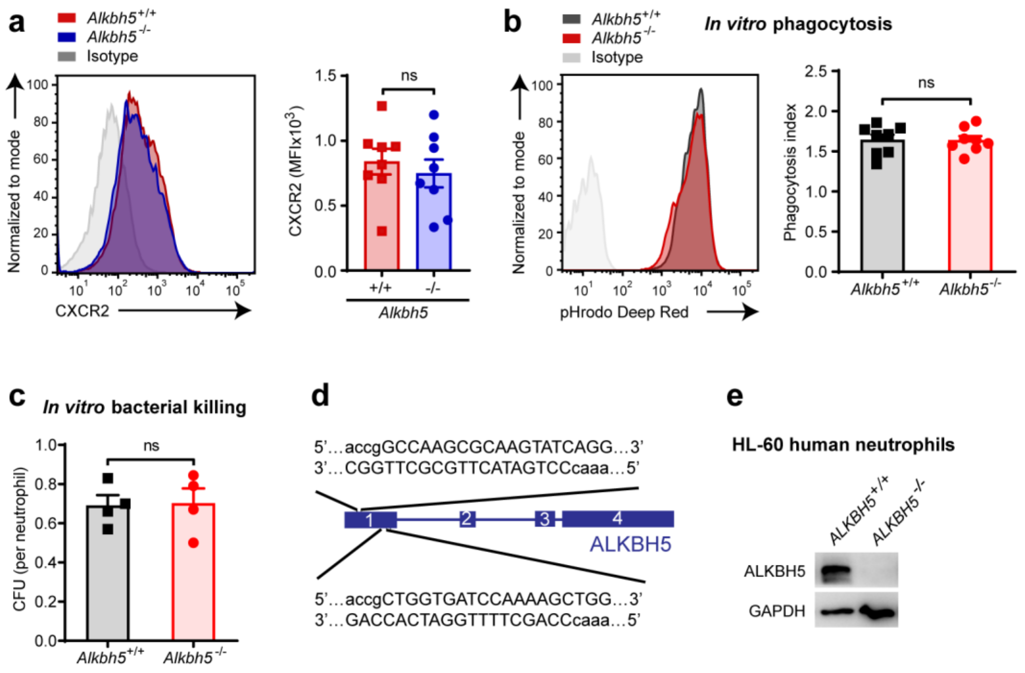

Figure. S7. ALKBH5 has no direct effect on the phagocytosis or bacteria-killing capability of neutrophils.

a. FACS analysis of protein expression of CXCR2 on cell-surface of peritoneal neutrophils from *Alkbh5*-deficient mice and WT littermates at 36h after mild CLP (*n*=8). **b.** *In vitro* phagocytosis assay of bone marrow neutrophils from normal *Alkbh5*-deficient mice and WT littermates. Phagocytosis index was expressed as the MFI of pHrodo Deep Red *E.coli* bioparticles engulfed by each neutrophil at 2h after infection (*n*=8). **c.** *In vitro* bacterial killing assay of bone marrow neutrophils from normal *Alkbh5*-deficient mice and WT littermates that were infected with live *E.coli* for 2h. *In vitro* bacterial killing capability was reflected by the residual CFU per neutrophil at 2h (*n*=4). **d-e.** Generation and identification of ALKBH5 knockout HL-60 cell line: CRISPR-cas9 strategy used to knockout the first exon of ALKBH5 in HL-60 cell line (**d**); Verification of ALKBH5 deletion in HL-60 single cell colonies by immunoblot analysis (**e**). All data are mean ± SEM of biologically independent samples. Data are representative of 3 or 8 independent experiments with similar results [(**a, b**) and (**e**)]. ns, not significant. Two-tailed unpaired Student's *t* test (**a-c**).

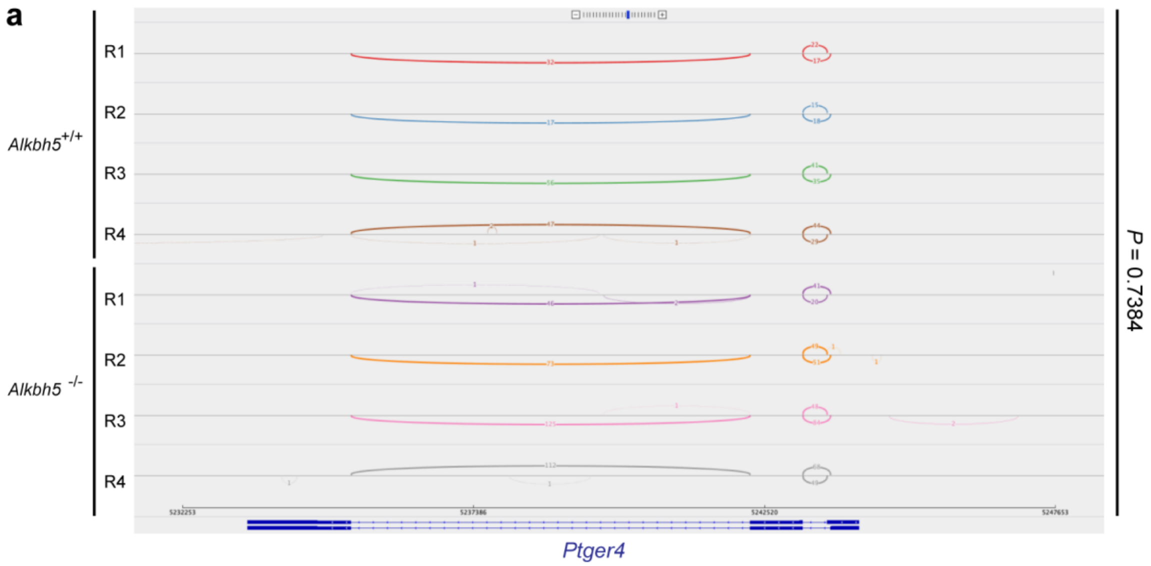

Figure. S8. ALKBH5 has no effect on the alternative splicing pattern of *Ptger4* or *Wnk1* mRNAs.

a. The alternative splicing pattern and *P* value of *Ptger4* mRNAs (as example) in peritoneal neutrophils of *Alkbh5*-deficient mice and WT littermates at 36h after mild CLP. Four biological replicates (R1 to R4). *P* value (*Ptger4*) = 0.7384; *P* value (*Wnk1*) = 1, indicating that ALKBH5 has no effect on the alternative splicing pattern of *Ptger4* or *Wnk1* mRNAs.

Table S1. m^6A peak abundance on mRNAs of indicated genes from m^6A-seq data.

Gene	Location	log_2(ES) in KO.Rep1	log_2(ES) in KO.Rep2	log_2(ES) in WT.Rep1	log_2(ES) in WT.Rep2	Replicate average_diff_ log_2 (ES)
Cxcr2	CDS	3.711	4.423	3.283	2.613	1.119
	Stop Codon	4.470	5.026	3.796	3.895	0.903
Nlrp12	3′UTR	1.807	2.010	0.778	1.115	0.962
Tnc	CDS	3.665	3.464	3.914	4.385	-0.585
	CDS	3.837	5.755	4.057	3.660	0.937
	3′UTR	1.206	1.285	-0.515	-0.415	1.710
Ptger4	CDS	1.906	2.007	1.475	0.804	0.817
	CDS	1.802	1.780	1.362	1.255	0.483
	CDS	2.775	3.255	2.573	2.123	0.667
	Stop Codon	1.429	1.436	0.928	0.951	0.493
	3′UTR	1.744	1.368	0.904	0.759	0.724
Wnk1	5′UTR	1.968	2.759	2.196	2.111	0.210
	CDS	5.795	7.213	5.231	5.802	0.988
	CDS	3.458	3.409	3.327	3.127	0.207
	CDS	2.551	2.548	2.152	2.717	0.115
	CDS	3.549	4.168	3.196	3.507	0.507
	Stop Codon	2.010	2.647	2.425	2.426	-0.097

Two independent biological replicates (Rep1 and Rep2) of m^6A-seq data (GSE127732). WT, wild-type; KO, *Alkbh5*-deficient. log_2(ES), log_2(enrichment score). Related to Fig. 6d.

Table S2. Primer sequences used for qRT-PCR.

Primers for mouse genes	Sequence (5′→3′)
Alkbh5 Forward	CGCGGTCATCAACGACTACC
Alkbh5 Reverse	ATGGGCTTGAACTGGAACTTG
Cxcr2 Forward	GGTGGGGAGTTCGTGTAGAA
Cxcr2 Reverse	CTACTACACAGGGATCAGGGC
Nlrp12 Forward	CCTCTTTGAGCCAGACGAAG
Nlrp12 Reverse	GCCCAGTCCAACATCACTTT
Ptger4 Forward	CGGTTCCGAGACAGCAAA
Ptger4 Reverse	CGGTTCGATCTAGGAATGG
Tnc Forward	TGTGTGCTTCGAAGGCTATG
Tnc Reverse	GCAGACACACTCGTTCTCCA
Wnk1 Forward	CTACAAAGGTCTGGACACCG
Wnk1 Reverse	ACTGTGGATTCCCAGGAATC
IL1r1 Forward	GTGCTACTGGGGCTCATTTGT
Il1r1 Reverse	GGAGTAAGAGGACACTTGCGAAT
Gapdh Forward	AGGTCGGTGTGAACGGATTTG
Gapdh Reverse	TGTAGACCATGTAGTTGAGGTCA
**Primers for human genes**	**Sequence (5′→3′)**
CXCR2 Forward	GCTCTGACTACCACCCAACC
CXCR2 Reverse	AGGACACCTCCAGAAGAGCA
NLRP12 Forward	CAGACTCCAGAAACTGTGG
NLRP12 Reverse	GCGTTGTTGGTCAGGTAAAG
PTGER4 Forward	CCGGCGGTGATGTTCATCTT
PTGER4 Reverse	CCCACATACCAGCGTGTAGAA
TNC Forward	ATTTGGGGACCGCAGAGAAGAA
TNC Reverse	TGTCCCCATATCTCCCCATCA
WNK1 Forward	CCTTTTCGTTCACGAATCCGAG
WNK1 Reverse	AACCGGGAGTGCTGCTCTGCTT
GAPDH Forward	TGCACCACCAACTGCTTAGC
GAPDH Reverse	GGCATGGACTGTGGTCATGAG
18S rRNA Forward	GTAACCCGTTGAACCCCATT
18S rRNA Reverse	CCATCCAATCGGTAGTAGCG

References

1 Zheng, G. *et al.* ALKBH5 is a mammalian RNA demethylase that impacts RNA metabolism and mouse fertility. *Mol Cell* **49**, 18-29, doi:10.1016/j.molcel.2012.10.015 (2013).

2 Liu, Y. *et al.* N (6)-methyladenosine RNA modification-mediated cellular metabolism rewiring inhibits viral replication. *Science* **365**, 1171-1176, doi:10.1126/science.aax4468 (2019).

3 Shen, Q. *et al.* Tet2 promotes pathogen infection-induced myelopoiesis through mRNA oxidation. *Nature* **554**, 123-127, doi:10.1038/nature25434 (2018).

4 Rittirsch, D., Huber-Lang, M. S., Flierl, M. A. & Ward, P. A. Immunodesign of experimental sepsis by cecal ligation and puncture. *Nat Protoc* **4**, 31-36, doi:10.1038/nprot.2008.214 (2009).

5 Alves-Filho, J. C. *et al.* Regulation of chemokine receptor by Toll-like receptor 2 is critical to neutrophil migration and resistance to polymicrobial sepsis. *Proceedings of the National Academy of Sciences of the United States of America* **106**, 4018-4023, doi:10.1073/pnas.0900196106 (2009).

6 Arandjelovic, S. *et al.* A noncanonical role for the engulfment gene ELMO1 in neutrophils that promotes inflammatory arthritis. *Nature immunology* **20**, 141-151, doi:10.1038/s41590-018-0293-x (2019).

7 Alves-Filho, J. C. *et al.* Interleukin-33 attenuates sepsis by enhancing neutrophil influx to the site of infection. *Nature medicine* **16**, 708-712, doi:10.1038/nm.2156 (2010).

8 Sakai, J. *et al.* Reactive oxygen species-induced actin glutathionylation controls actin dynamics in neutrophils. *Immunity* **37**, 1037-1049, doi:10.1016/j.immuni.2012.08.017 (2012).

9 Li, Y. *et al.* Targeted deletion of tumor suppressor PTEN augments neutrophil function and enhances host defense in neutropenia-associated pneumonia. *Blood* **113**, 4930-4941, doi:10.1182/blood-2008-06-161414 (2009).

10 Chen, Y. *et al.* ALKBH5 suppresses malignancy of hepatocellular carcinoma via m(6)A-guided epigenetic inhibition of LYPD1. *Mol Cancer* **19**, 123, doi:10.1186/s12943-020-01239-w (2020).
